# A complementary pair of enantioselective switchable organocatalysts†
†Electronic supplementary information (ESI) available. See DOI: 10.1039/c7sc02462b



**DOI:** 10.1039/c7sc02462b

**Published:** 2017-08-23

**Authors:** Guillaume De Bo, David A. Leigh, Charlie T. McTernan, Shoufeng Wang

**Affiliations:** a School of Chemistry , University of Manchester , Oxford Road , Manchester , M13 9PL , UK . Email: david.leigh@manchester.ac.uk

## Abstract

A pair of enantioselective switchable bifunctional catalysts are shown to promote a range of conjugate addition reactions in up to 95 : 5 e.r. and 95% conversion. Each catalyst can be switched OFF using conditions that switch the other catalyst ON. Catalyst ON : OFF ratios of up to 98 : 2 and 1 : 99 were achieved, with a ratio of reaction rates of up to 16 : 1 between the ON and OFF states, maintained over complete ON–OFF–ON and OFF–ON–OFF cycles. However, simultaneous operation of the catalyst pair in the same reaction vessel, which in principle could allow product handedness to be switched by simple *E*–*Z* isomerisation of the catalyst pair, was unsuccessful. In this first generation complementary pair of enantioselective switchable organocatalysts, the OFF state of one catalyst inhibits the ON state of the other.

## Abstract 2

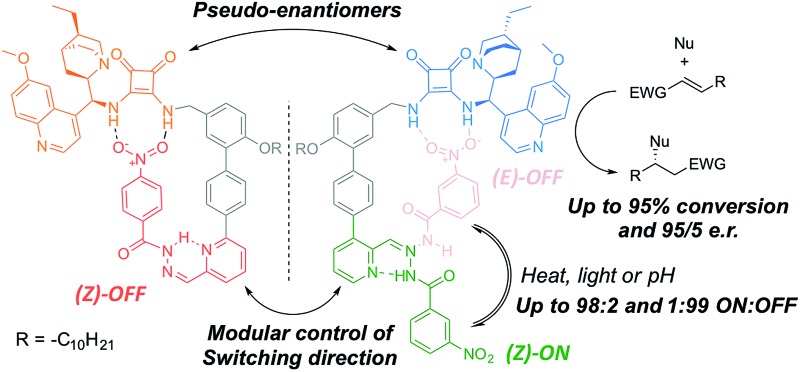
A pair of enantioselective switchable bifunctional catalysts are shown to promote a range of conjugate addition reactions in up to 95 : 5 e.r. and 95% conversion.

## Introduction

Trigger effects and feedback loops are key processes used to control enzyme activity in cells.^1^ They help to maintain homeostasis and enable lengthy reaction pathways to be carried out in complex chemical environments. Such switchable catalytic behaviour is mimicked in an array of artificial systems^2^ in order to modulate rate,^3^ reaction pathway^4^ and, somewhat less frequently, stereoselectivity.^5^ However, state-of-the-art catalytic systems are often sensitive to structural modification and their incorporation into molecular switches and other devices often leads to significant erosion of selectivity or activity.^4c,5d^ This is especially true for bifunctional organocatalysts,^6*a*^ which typically require the cooperation of a (chiral) base and a hydrogen-bond donor.^6*b*^ Here we demonstrate a modular approach to enantioselective switchable bifunctional organocatalysts that provides a high degree of control over catalyst activity and stereoselectivity with little loss of efficacy of organocatalytic function. By judicious choice of the regiochemistry of attachment of the switching unit, a complementary pair of enantioselective catalysts was prepared using this approach, in which each catalyst could be switched ON under conditions that switched the other catalyst OFF.

**Scheme 1:**
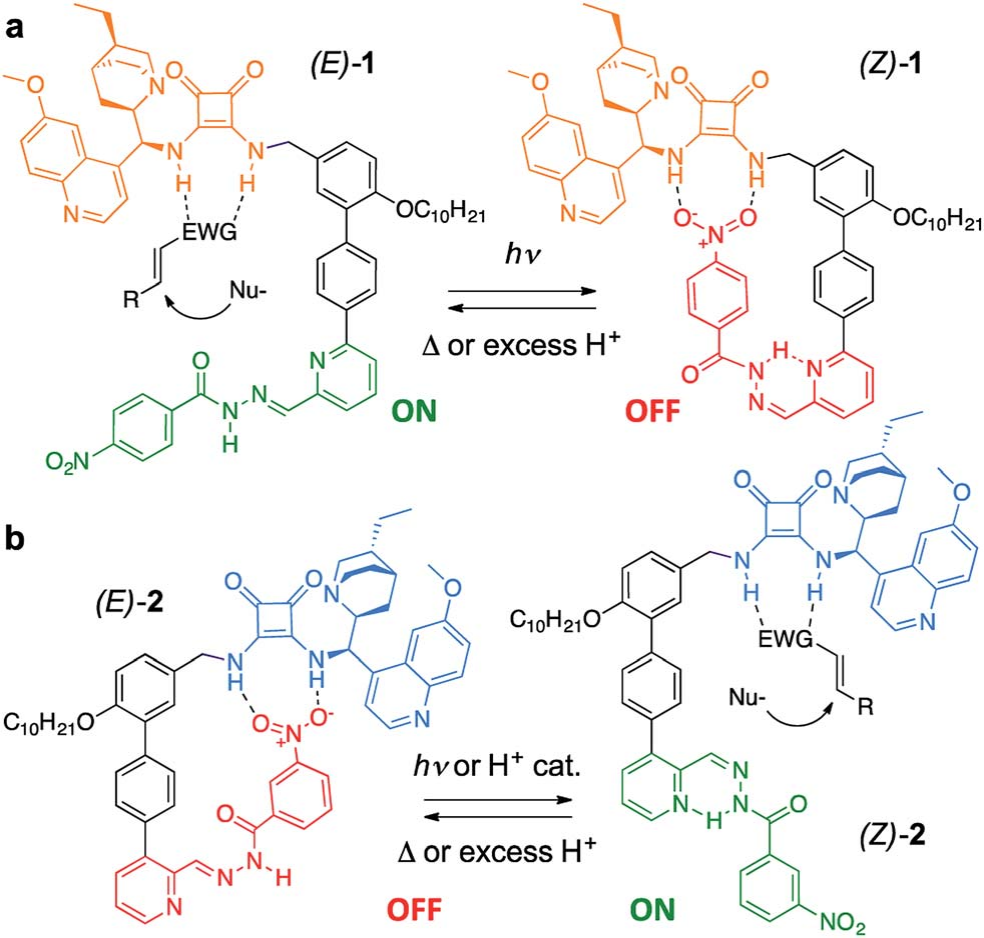
A pair of enantioselective switchable bifunctional organocatalysts.

The catalyst pair is based on a bifunctional cinchona alkaloid-squaramide motif as the active catalyst,^7^ with a hydrogen bond acceptor, nitrobenzene, as an additional potentially inhibiting unit (Scheme 1). The *E*/*Z* isomerisation of a pyridyl-acyl hydrazone switch^8,9^ is used to control the activity of the catalyst. In **1**, the catalytically inactive OFF state is generated by operating the switch with UV light, bringing the nitro group into close proximity with the squaramide (Scheme 1a). By changing the regiochemistry of the pyridine ring (**2**), the same stimulus can be used to turn catalyst **2** ON through the equivalent isomerisation process forcing the nitro group away from the squaramide (Scheme 1b). The resulting pair of enantioselective switchable bifunctional organocatalysts can be switched between states with various stimuli (light, heat or pH), giving ON : OFF ratios of up to 98 : 2 and 1 : 99. In their ON state each catalyst promotes Michael additions with an enantiomeric ratio (e.r.) of up to 95 : 5.

## Results and discussion

**Scheme 2:**
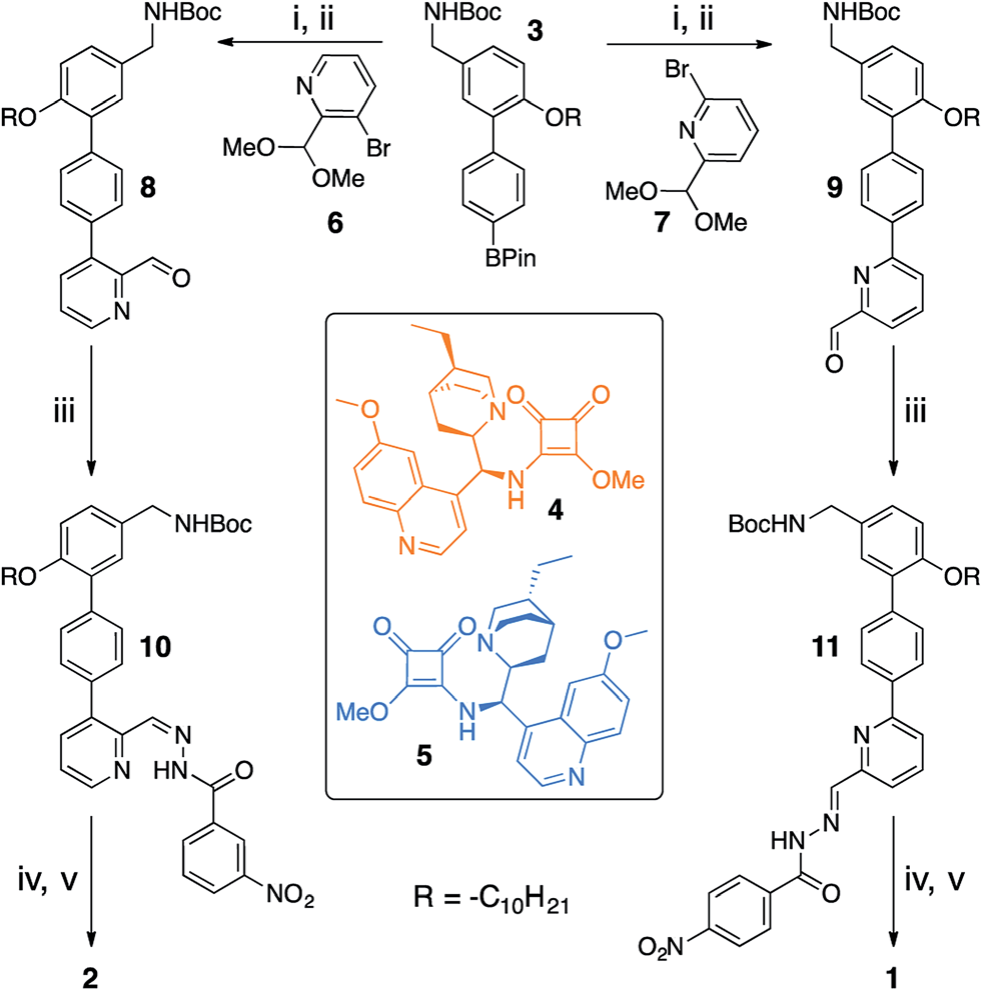
Modular assembly of enantioselective switchable catalysts **1** and **2**. Reagents and conditions: (i) **6** or **7**, Pd(PPh_3_)_4_, THF, 2 M Na_2_CO_3_, 60 °C, 4 days. (ii) *p*-Toluenesulfonic acid, THF, H_2_O, 50 °C, 3 h. (iii) 3- or 4-Nitrobenzohydrazide, aniline, CH_2_Cl_2_, r.t., 16 h. (iv) CF_3_CO_2_H, CH_2_Cl_2_, r.t., 1 h. (v) **4** or **5**, DMF, Et_3_N, MeOH, r.t., 16 h.

Switchable catalysts **1** and **2** were assembled (Scheme 2) around a biphenyl unit **3** featuring (i) an *n*-decyl chain to ensure sufficient organic solvent solubility of the final catalyst, (ii) a protected amine to anchor the catalytic units **4** or **5**, and (iii) a boronic ester through which to attach the switching unit. Complementary switching behaviour was achieved by coupling **3** with 2,3-substituted bromopyridine **6** or its 2,6-isomer **7**. The squaramide inhibiting unit was installed by condensing a nitrobenzohydrazide with aldehydes **8** or **9** to form hydrazones **10** and **11**. Finally, amine deprotection followed by addition of hydroquinine-derived mono-squaramide **4** or hydroquinidine-derived mono-squaramide **5** furnished catalysts **1** and **2**, respectively. Catalysts **1** and **2** were synthesised in 14 steps in overall yields of 18% and 12%, respectively (see ESI† for full synthetic procedures and characterisation data).

With **1** and **2** in hand our attention turned to switching between the *E*- and *Z*-states of the catalyst pair. Catalyst **1** was typically formed in a 80 : 20 *E* : *Z* ratio from Scheme 2, and optimised light-switching conditions provided a 21 : 79 *E* : *Z* ratio at 395 nm (Table 1, entry 1 and Table S1†). The most effective conditions for *Z*-to-*E* isomerisation proved to be using CF_3_CO_2_H in ethyl acetate at 50 °C, which afforded 98 : 2 ratio of *E* : *Z*-**1** (Table 1, entry 3 and Table S2†).

**Table 1:** *E*-to-*Z* and *Z*-to-*E* switching conditions for **1** and **2**. See ESI and Tables S1 and S2 for details

Entry	Catalyst	Conditions	*E* : *Z*
1	**1**	CH_2_Cl_2_ : EtOAc 1 : 4, 395 nm, 700 mW, 20 min, 0.3 mg ml^–1^	21 : 79
2	**1**	DMF, 90 °C, 5 h, 0.2 mg ml^–1^	90 : 10
3	**1**	EtOAc, 0.1% CF_3_CO_2_H, 60 min, 50 °C, 0.02 mg ml^–1^	98 : 2
4	**2**	CH_2_Cl_2_ : EtOAc 1 : 4, 395 nm, 700 mW, 20 min, 0.3 mg ml^–1^	21 : 79
5	**2**	EtOAc, 0.1% CF_3_CO_2_H, 60 min, 50 °C, 0.02 mg ml^–1^	99 : 1
6	**2**	CHCl_3_, 0.25 eq. CF_3_CO_2_H, 24 h, 40 °C, 0.1 mg ml^–1^	5 : 95

As with catalyst **1**, catalyst **2** formed a 21 : 79 *E* : *Z* mixture under irradiation at 395 nm (entry 4, Table 1). A 99 : 1 *E* : *Z* ratio was reached upon heating **2** with 0.1% CF_3_CO_2_H in EtOAc (entry 5). Somewhat surprisingly, 0.25 equiv. CF_3_CO_2_H in CHCl_3_ promoted *E*-to-*Z* isomerisation to give a 5 : 95 *E* : *Z* ratio of isomers (entry 6), although these conditions proved ineffective with catalyst **1**. The ability to use either light or one of two different acid-based conditions to promote switching augurs well for operating the catalytic groups in more complex settings.

Bifunctional cinchona-squaramide catalysts are known to promote a range of conjugate addition reactions,^10^ so we focused on examining this reaction class with **1** and **2** (Table 2). The addition of malonitrile or masked thiol to chalcone derivatives was catalysed at low catalyst loadings with good conversions and e.r. (Table 2, entries 1, 2 and 5), as was the addition of 1,3-diphenyl-1,3-propandione or diethyl malonate to β-nitrostyrene (Table 2, entries 3 and 4). Masked acyl cyanides could be generated with excellent conversions and very good e.r. (Table 2, entry 6).^11^ Overall, the conversions and e.r. are similar to previously reported bifunctional organocatalysts (Table 2, entries 1–5), despite the switching process leaving some catalyst in the OFF state (catalyst **1** used as 98 : 2 *E* : *Z*; catalyst **2** used as 5 : 95 *E* : *Z*). The results show that catalytic motifs can be incorporated into more complex switching structures without significantly impairing catalytic performance.

**Table 2:** Catalytic activity of switchable catalysts **1** and **2** compared to previously reported data for related but non-switchable organocatalysts. Conditions: nucleophile (1 equiv.), electrophile (10 equiv.), catalyst (5 mol%), CDCl_3_, r.t., 24 h, except entries 2 (50 °C, 48 h, d_8_-toluene), 4 (1 week, catalyst (25 mol%)) and 6 (CD_2_Cl_2_). The product of entry 2 was isolated as a single diastereomer (determined by ^1^H NMR and HPLC) with catalyst **1** producing (*S*,*R*,*R*) as the major enantiomer. Enantiomers assigned by reference to literature

Entry	Reagents	Product^*a*^	Conv. (*S* : *R*), **1**	Conv. (*S* : *R*), **2**	Prev. report^*b*^ (*S* : *R*)
1	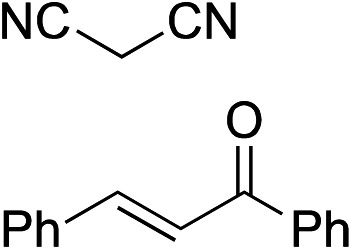	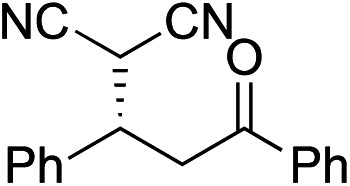	91% (83 : 17)	90% (23 : 77)	82% (95 : 5)^10*c*^
2	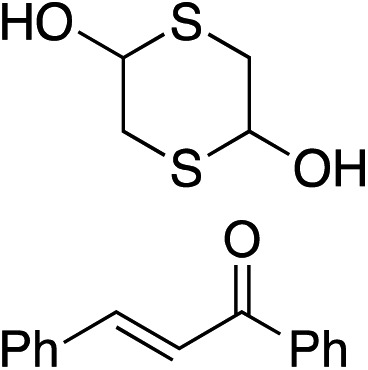	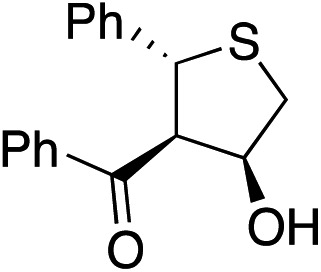	93% (25 : 75)	94% (70 : 30)	81% (94 : 6)^10*d*^
3	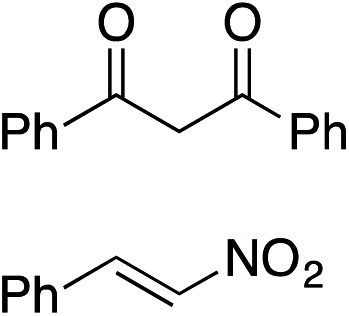	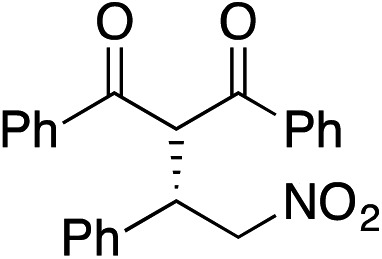	81% (91 : 9)	90% (10 : 90)	89% (95 : 5)^10f,h^
4	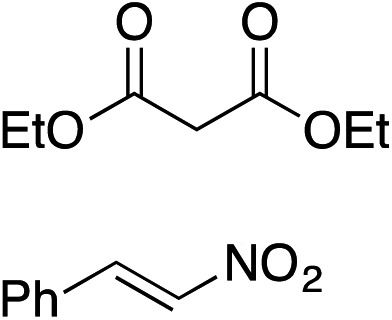	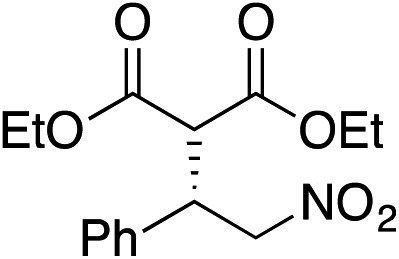	88% (89 : 11)	70% (20 : 80)	83% (95 : 5)^10*h*^
5	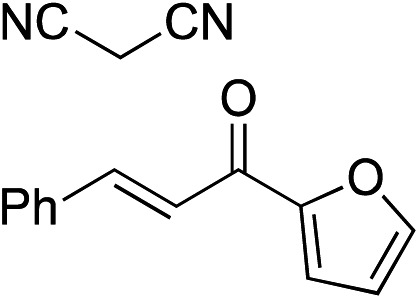	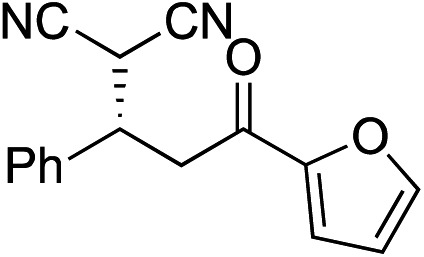	95% (95 : 5)	93% (7 : 93)	96% (93 : 7)^10*e*^
6	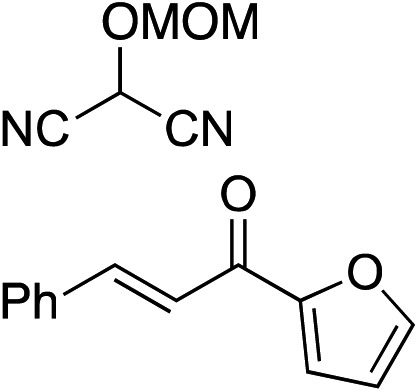	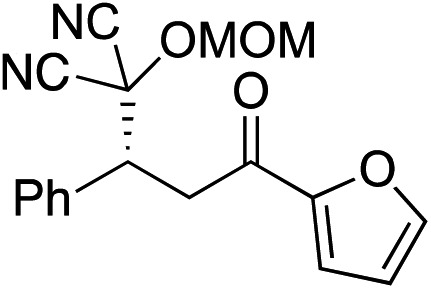	95% (87 : 13)	95% (16 : 84)	—

^*a*^Products of catalyst **1** depicted.

^*b*^Catalyst structures and reaction conditions are shown in Table S3, ESI. MOM = methoxymethyl. In all cases catalyst **1** was used as 98 : 2 *E* : *Z* and catalyst **2** as 1 : 99 *E* : *Z*.

**Fig. 1:**
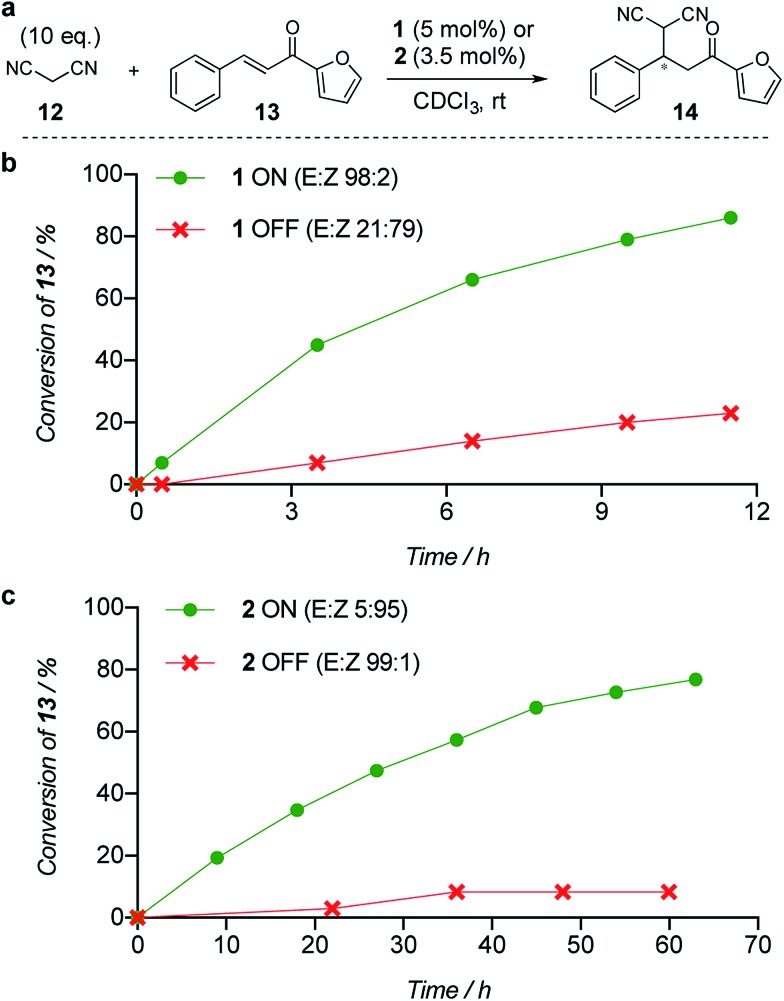
Relative reactivity of ON and OFF states of (b) catalyst **1** and (c) catalyst **2** towards the addition of malonitrile (**12**) to chalcone (**13**). Solid lines are a guide to the eye.

The limiting factor that determines the efficacy of turning catalysis ON and OFF with a switchable catalyst is generally not how effective the ON state is as a catalyst, but rather the lack of catalysis by the OFF state.^2*a*^ Often the OFF state still catalyses the reaction to some extent, which is compounded if the switching leaves even a small amount of the ON state of the catalyst present.^2*a*^ We used the formation of **14** by addition of malonitrile (**12**) to chalcone **13** (Fig. 1a) to investigate the relative reactivity of the ON and OFF states of each member of the catalyst pair (Fig. 1b and c). The reactions were monitored by ^1^H NMR and, as expected from the imperfect photostationary state (*E* : *Z* = 21 : 79 for the OFF state of **1**), a 6 : 1 ON : OFF ratio of initial rates was observed when using 5 mol% of catalyst **1** (Fig. 1b). With a 3.5 mol% loading of catalyst **2** (*E* : *Z* = 99 : 1 for the OFF state), an ON : OFF ratio of 16 : 1, excellent for the current generation of artificial switchable catalysts, was achieved (Fig. 1c). Finally we investigated the switching of catalysis ON and OFF during the chemical reaction of **12** and **13** (see ESI†). A full switching cycle was carried out starting from either the ON or OFF state of catalyst **2** (3.5 mol%, initial *E* : *Z* ratio 99 : 1 (ON) or 2 : 98 (OFF)). After 6 h the *E*-to-*Z* (or *Z*-to-*E*) stimulus was applied (0.1% CF_3_CO_2_H, 60 min, 50 °C or 395 nm, 700 mW, 20 min) and after 24 h (for initially OFF **2**) or 21 h (for initially ON **2**) the opposing stimulus was applied reverting catalyst **2** to its initial state. In both cases good control of reaction rate over the entire cycle was achieved, without compromising the stereoselectivity of product formation (see ESI†).

The ultimate goal of a complementary pair of switchable enantioselective catalysts is the enticing prospect of being able to operate them simultaneously in the same reaction vessel so that the handedness of the product produced by the catalysis can be controlled by switching OFF one catalyst while the same stimulus switches ON the other. Our attempts to achieve this with **1** and **2** proved unsuccessful, with the organocatalysis remaining OFF when either stimulus was applied. Bifunctional cinchona-squaramide catalysts have a tendency to aggregate^12^ and for **1** and **2** it appears that the OFF state of one catalyst binds to the ON state of the other catalyst, preventing catalysis by either state of either member of the catalyst pair. Inhibiting association between the catalysts must be an important consideration in future designs of complementary pairs of switchable enantioselective catalysts.

## Conclusions

A complementary pair of switchable enantioselective catalysts, **1** and **2**, were prepared, each capable of catalyzing Michael additions of carbon and sulfur nucleophiles with chalcones in 70–95% yield with good stereoselectivity (up to 95 : 5 e.r.). The switchable catalysts have similar activities to previously reported non-switchable catalysts and so incorporating the bifunctional organocatalytic groups into a more complex, switchable, molecular structure does not significantly impact on the intrinsic efficacy of the catalytic groups. The two switchable catalysts generate opposite enantiomers, so both hands of products can be accessed, and a single stimulus switches one member of the catalyst pair OFF and the other ON. Photochemical or acid-catalysed isomerisation provides excellent *E* : *Z* ratios in each switching direction, and significant differences in ON *versus* OFF rates of catalysis were obtained, which were maintained throughout full ON–OFF–ON and OFF–ON–OFF cycles. Future work will focus on the design of systems where the complementary pair of catalysts can be operated simultaneously in the same reaction vessel.

## Conflicts of interest

There are no conflicts to declare.
