# Supplementary material for: A complementary pair of enantioselective switchable organocatalysts
Source: Chem Sci. 2017 Aug 23;8(10):7077–81. doi: 10.1039/c7sc02462b (PMC5637462; doi:10.1039/c7sc02462b)
Supplement: Supplementary file 1 [file SC-008-C7SC02462B-s001.pdf]

Electronic Supplementary Material (ESI) for Chemical Science.
This journal is © The Royal Society of Chemistry 2017

Supporting Information

A Complementary Pair of Enantioselective Switchable Organocatalysts

Guillaume De Bo, David A. Leigh,* Charlie T. McTernan, Shoufeng Wang

School of Chemistry, University of Manchester, Oxford Road, Manchester, M13 9PL, United Kingdom

Email: david.leigh@manchester.ac.uk

Table of contents

1. General Methods……S3
2. Synthesis……S4
3. Catalytic tests……S14
4. Optimization of light switching……S17
5. Optimization of heat switching……S19
6. Literature Catalysis Examples……S20
7. *in situ* Switching experiments……S21
8. HPLC traces……S22
9. NMR Spectra……S26
10. References……S40

1. General Methods

Unless stated otherwise, reagents were obtained from commercial sources and used without purification. Anhydrous THF (HPLC grade, Fischer scientific), CH_2Cl_2 (HPLC grade, Fischer scientific), DMF (Peptide synthesis grade, Merck) and PhMe (> 99%, Fischer scientific) were obtained by passing the solvent through an activated alumina column on a Phoenix SDS (solvent drying system; JC Meyer Solvent Systems, CA, USA). ^1H NMR spectra were recorded on a Bruker Avance III instrument with an Oxford AS600 magnet equipped with a cryoprobe [5mm CPDCH ^{13}C-^1H/D] (600 MHz). Chemical shifts are reported in parts per million (ppm) from high to low frequency using the residual solvent peak as the internal reference ($CDCl_3$ = 7.26 ppm, CD_2Cl_2 = 5.32 ppm, CD_3OD = 3.31 ppm, d_6-Acetone = 2.05 ppm and d_6-DMSO = 2.50 ppm). All ^1H resonances are reported to the nearest 0.01 ppm. The multiplicity of ^1H signals are indicated as: s = singlet; d = doublet; t = triplet; quint = quintet; m = multiplet; br = broad; or combinations of thereof. Coupling constants (J) are quoted in Hz and reported to the nearest 0.1 Hz. ^{13}C NMR spectra were recorded on the same spectrometer with the central resonance of the solvent peak as the internal reference ($CDCl_3$ = 77.16 ppm, CD_2Cl_2 = 54.00 ppm, CD_3OD = 49.00 ppm, d_6-Acetone = 29.84 ppm and d_6-DMSO = 39.52 ppm). ^{19}F NMR spectra were recorded on a Bruker Avance III instrument (376 MHz). DEPT, COSY, HSQC and HMBC experiments were used to aid structural determination and spectral assignment. Flash column chromatography was carried out using Silica 60 Å (particle size 40–63 µm, Sigma Aldrich, UK) as the stationary phase. Preparative TLC was performed using PLC 20 × 20 cm, 60 F254 Prep plates (Merck, of various thicknesses). TLC was performed on precoated silica gel plates (0.25 mm thick, 60 F254, Merck, Germany) and visualized using both short and long waved ultraviolet light in combination with standard laboratory stains (acidic potassium permanganate, acidic ammonium molybdate and ninhydrin). Low resolution ESI mass spectrometry was performed with a Thermo Scientific LCQ Fleet Ion Trap Mass Spectrometer or an Advion Compact Mass Spectrometer (CMS). High-resolution mass spectrometry was carried out by the EPSRC National Mass Spectrometry Service Centre (Swansea, UK) or by the departmental service. Enantiomeric ratios were determined by HPLC on an Agilent 1260 Infinity system with UV detection at 210, 250 or 254 nm. A Chiralpak IA or IC (5 µm Particle size, 250×4.6 mm, Diacel Corporation) column with hexane/2-propanol (90/10) as eluent (1 ml/min flow-rate) was used for separations unless otherwise stated. Solutions were irradiated in 3.5 mL quartz cuvettes (10 × 10 mm) with a 395 nm LED source (FWHM = 15 nm, ThorLabs M395L4). Typically, solutions were irradiated for 20 minutes with an irradiance of 700 $mW\cdot cm^{-2}$, as measured using a power meter (ThorLabs PM100D equipped with an S302C thermal sensor).

Abbreviations: DMF, dimethyl formamide. DMSO, dimethyl sulfoxide, Pd(dppf), {[1,1′-bis(diphenylphosphino)ferrocene] dichloropalladium(II), complex with CH_2Cl_2} . PE, petroleum ether. HRMS (ESI^+), high resolution electrospray mass spectrometry. MS (ESI^+), low resolution electrospray mass spectrometry. *p*TSA, *para*-toluenesulfonic acid. DIPEA, diisopropylethylamine. THF, tetrahydrofuran. Phenyl triflimide, *N*-Phenyl-bis(trifluoromethanesulfonimide). Boc_2O, Di-*tert*-butyl dicarbonate.

2. Synthesis of 1 and 2

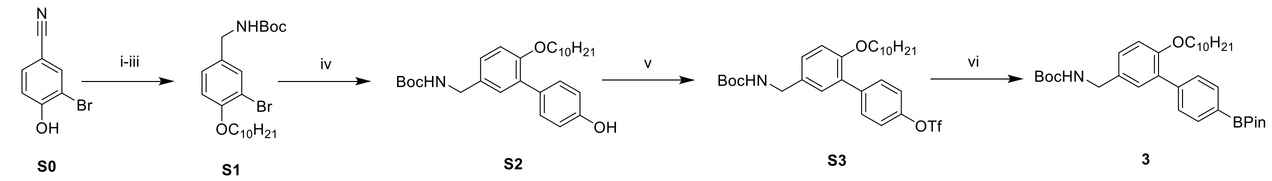

Reagents and conditions. i) 1-Bromodecane, Cs_2CO_3, MeCN, reflux, 16 h. ii) BH_3.THF, THF, reflux, 16 h. iii) Boc_2O, CH_2Cl_2, DIPEA r.t., 1 h. iv) 4-Hydroxyphenylboronic acid, Pd(dppf), THF, 2 M Na_2CO_3, 16 h. v) Phenyl triflimide, DIPEA, DMF, r.t., 16 h. vi) Bispinocolatodiboron, Pd(dppf), KOAc, dioxane, 100 °C, 16 h.

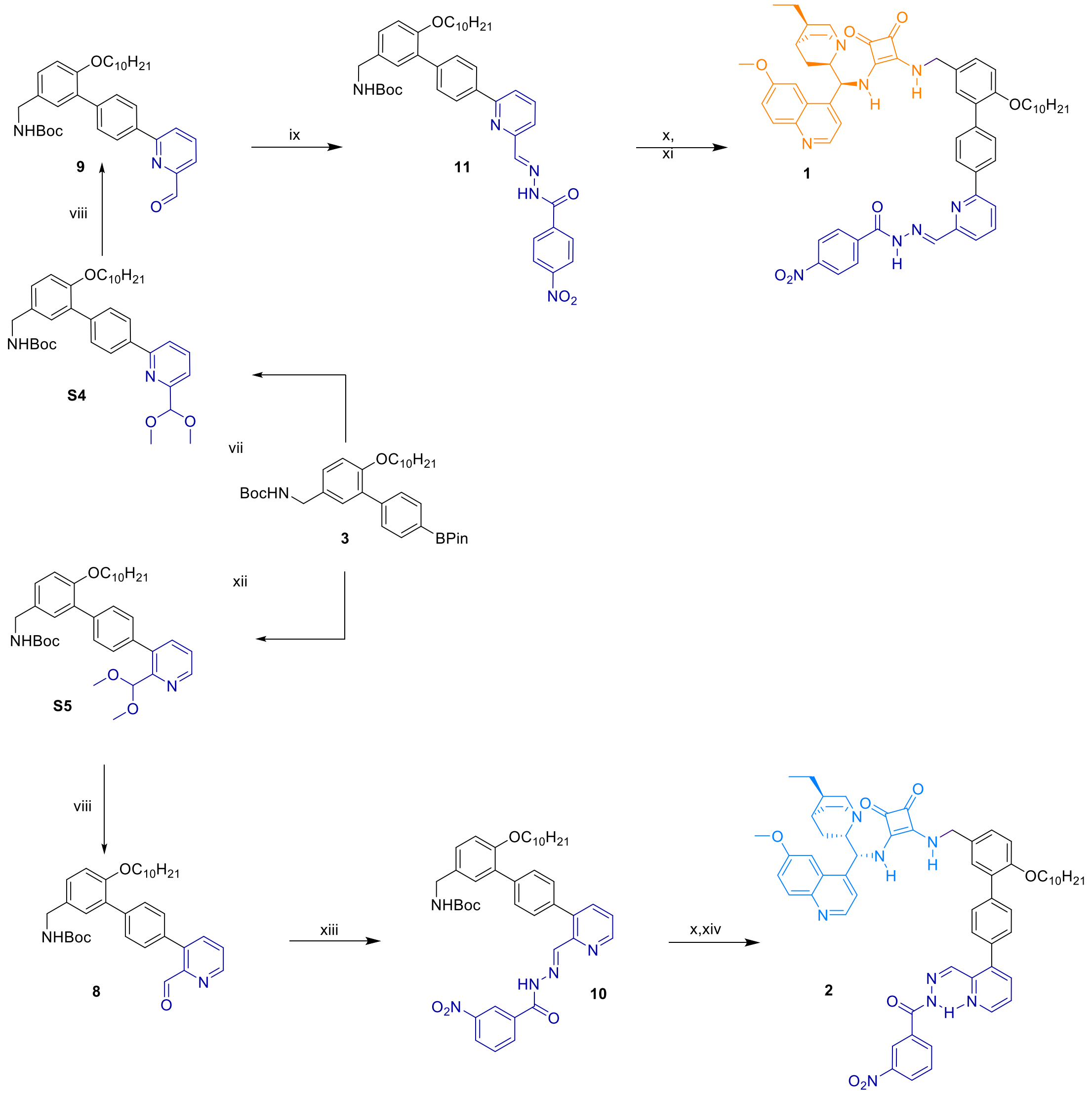

Reagents and conditions: vii) 2-Bromo-6-(dimethoxymethyl)pyridine (**7**), $Pd(PPh_3)_4$, THF, 2 M Na_2CO_3, 60 °C, 4 d. viii) *p*TSA, THF, H_2O, 50 °C, 3 h. ix) 4-Nitrobenzohydrazide (**21**), aniline, CH_2Cl_2, r.t., 16 h. x) TFA, CH_2Cl_2, r.t., 1 h. xi) 3-Amino-4-(((*S*)-((1*S*,2*R*,4*S*,5*R*)-5-ethylquinuclidin-2-yl)(7-methoxynaphthalen-1-yl)methyl)amino)cyclobut-3-ene-1,2-dione (**4**), DMF, Et_3N, MeOH, r.t., 16 h.

xii) 3-Bromo-2-(dimethoxymethyl)pyridine (**6**), $Pd(PPh_3)_4$, THF, 2 M Na_2CO_3, 60 °C, 4 d. xiii) 3-Nitrobenzohydrazide (**22**), aniline, CH_2Cl_2, r.t., 16 h. xiv) 3-amino-4-(((*R*)-((1*S*,2*S*,4*S*,5*R*)-5-ethylquinuclidin-2-yl)(7-methoxynaphthalen-1-yl)methyl)amino)cyclobut-3-ene-1,2-dione (**5**), DMF, Et_3N, MeOH, r.t., 16 h.

7,[S1] **6**,[S2] **4**,[S3] **5**,[S3] **21**[S4] and **22**[S5] were synthesized according to literature procedures.

S1. *tert*-Butyl (3-bromo-4-(decyloxy)benzyl)carbamate

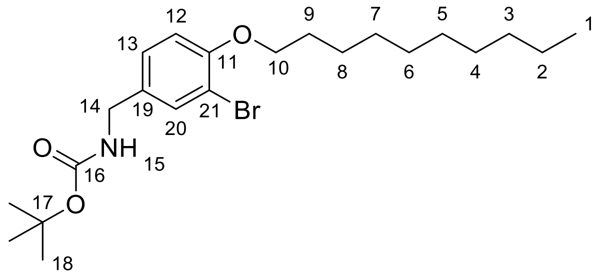

To a stirred suspension of 3-bromo-4-hydroxybenzonitrile (5.00 g, 25.2 mmol) and caesium carbonate (8.21 g, 25.2 mmol) in MeCN (100 ml) was added 1-bromodecane (5.53 g, 25.0 mmol). The suspension was then heated to reflux for 16 h then the reaction was cooled to r.t., and partitioned between Et_2O (500 ml) and 1 M $NaOH_{(aq)}$ (500 ml). The organic phase was washed with 1 M $NaOH_{(aq)}$ (500 ml), dried over $MgSO_4$, filtered and concentrated *in vacuo* to afford the crude product. The crude product was then dissolved in THF (50 ml) and cooled to 0 °C. To this stirred solution, BH_3.THF (1 M in THF, 40 ml) was added dropwise. The reaction was heated to reflux for 16 h then cooled to 0 °C and quenched with H_2O (2 ml) followed by 1 M $NaOH_{(aq)}$ (2 ml). The solvent was removed *in vacuo* and the residue dissolved in CH_2Cl_2 (200 ml). This solution was then washed with 2 M $NaOH_{(aq)}$ (200 ml), dried over $MgSO_4$, filtered and concentrated *in vacuo* to afford the crude product. The crude was then dissolved in CH_2Cl_2 (200 ml) and diisopropylethylamine (7.5 ml). To this was added di-*tert*-butyl dicarbonate (4.20 g, 19.2 mmol) all at once. The reaction was stirred at r.t. for 1 h and the solvent removed *in vacuo*. Imidazole (3.00 g, 44.0 mmol) was added to the residue, which was then dissolved in EtOH (75 ml), and stirred at r.t. for 5 min. The solvent removed *in vacuo* and the residue partitioned between $CHCl_3$ and 1 M $HCl_{(aq)}$. The organic phase was dried over $MgSO_4$, filtered, and the solvent removed *in vacuo* to afford the crude product. Purification by flash column chromatography [EtOAc:PE 7:3] afforded ***S1*** (5.51 g, 12.1 mmol, 50%) as a pale yellow oil.

^{1}H NMR (600 MHz, $CDCl_3$) δ: 7.45 (1H, s, H_{20}), 7.16 (1H, d, J = 7.9 Hz, H_{13}), 6.82 (1H, d, J = 8.2 Hz, H_{12}), 4.79 (1H, s, br, H_{15}), 4.20 (2H, d, J = 6.0 Hz, H_{14}), 4.00 (3H, t, J = 6.9 Hz, H_{10}), 1.85–1.72 (2H, m, H_9), 1.51–1.41 (11H, m, $H_8 + H_{18}$), 1.39–1.22 (12H, m, H_7–H_2), 0.88 (3H, t, J = 7.3 Hz, H_1); **^{13}C NMR** (150 MHz, $CDCl_3$) δ: 155.6, 154.8, 132.4, 132.3, 127.6, 113.2, 112.3, 79.6, 69.3, 43.7 ,31.9, 29.6, 29.6, 29.3, 29.1, 28.4, 26.0, 22.7, 14.2; **MS** (ESI^+): **m/z =** 442.2 [100, $(M+H)^+$], 464.3 [35, $(M+Na)^+$]; **HRMS** (ESI^+): $[C_{22}H_{40}BrN_2O_3]^+$ predicted 459.2217, found 459.2207 (Δ − 2.1 ppm).

S2. *tert*-Butyl ((6-(decyloxy)-4'-hydroxy-[1,1'-biphenyl]-3-yl)methyl)carbamate

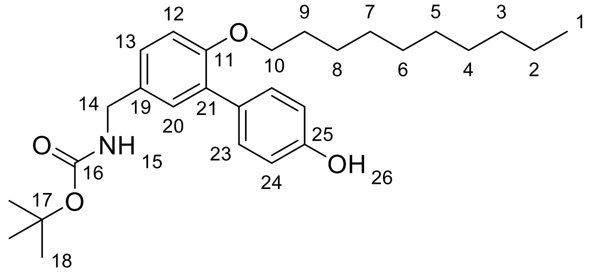

Bromide **S1** (6.00 g, 13.6 mmol) was dissolved in DMF (60 ml) and 2 M $Na_2CO_{3(aq)}$ (20 ml). The reaction was sparged with argon for 15 minutes, then 4-hydroxyphenylboronic acid (3.50 g, 25.4 mmol) and Pd(dppf) (800 mg, 0.980 mmol) were added. The reaction was heated under argon to 80 °C for 16 h then cooled to r.t. and the solvent removed *in vacuo*. The residue was partitioned between CH_2Cl_2 and $NaCl_{(sat)}$, and the aqueous phase washed once with CH_2Cl_2. The combined organic layers were dried over $MgSO_4$, filtered, and concentrated *in vacuo*. The crude residue was purified by flash column chromatography [EtOAc:PE 1:19 grading to 1:4] to furnish **S2** (5.97 g, 13.1 mmol, 96%) as a pale yellow oil.

^{1}H NMR (600 MHz, $CDCl_3$) δ: 7.41 (2H, d, J = 8.5 Hz, H_{23}), 7.20 (1H, s, H_{20}), 7.17 (1H, d, J = 8.1 Hz, H_{13}), 6.90 (1H, d, J = 8.4 Hz, H_{12}), 6.85 (2H, d, J = 8.5 Hz, H_{24}), 5.01 (1H, s, H_{26}), 4.80 (1H, s, br, H_{15}), 4.28 (2H, d, J = 4.9 Hz, H_{14}), 3.92 (3H, t, J = 6.7 Hz, H_{10}), 1.72–1.68 (2H, m, H_9), 1.46 (9H, s, H_{18}), 1.40–1.34 (2H, m, H_8), 1.31–1.20 (12H, m, H_7–H_2), 0.88 (3H, t, J = 7.1 Hz, H_1); **^{13}C NMR** (150 MHz, $CDCl_3$) δ: 156.0, 155.5, 154.8, 131.0, 131.0, 131.0, 130.7, 130.1, 129.8, 127.5, 114.9, 112.8, 79.6, 68.7, 44.4, 32.0, 29.7, 29.7, 29.5, 29.4, 28.6, 26.2, 22.8, 14.3; **MS** (ESI$^+$): **m/z =** 456.3 [100, $(M+H)^+$], 478.3 [25, $(M+Na)^+$]; **HRMS** (ESI$^+$): $[C_{28}H_{42}NO_4]^+$ predicted 456.3108, found 456.3102 (Δ − 1.4 ppm).

S3. 5'-(((*tert*-Butoxycarbonyl)amino)methyl)-2'-(decyloxy)-[1,1'-biphenyl]-4-yl trifluoromethanesulfonate

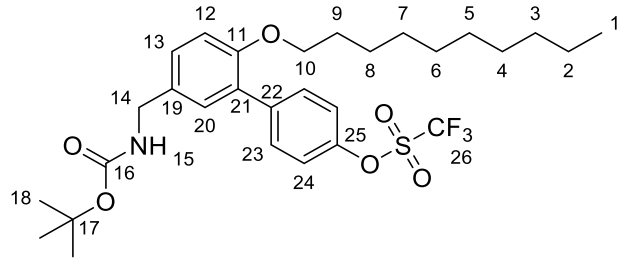

To a stirred solution of **S2** (5.50 g, 12.1 mmol) in DMF (150 ml) and diisopropylethylamine (8 ml) was added *N*-phenyl-bis(trifluoromethanesulfonimide) (5.00 g, 14.0 mmol). The reaction was stirred at r.t. for 16 h before the solvent was removed *in vacuo*. The residue was partitioned between CH_2Cl_2 (200 ml) and 2 M $HCl_{(aq)}$ (200 ml). The organic phase was washed with 2 M $NaOH_{(aq)}$ (200 ml) then $LiCl_{(sat)}$, dried over $MgSO_4$, filtered, and concentrated *in vacuo*. The crude product was purified by flash column chromatography [EtOAc:PE 1:9] to furnish ***S3*** (6.72 g, 11.8 mmol, 97%) as a yellow oil.

^{1}H NMR (600 MHz, CD_2Cl_2) δ: 7.63 (2H, d, J = 8.8 Hz, H_{23}), 7.35 (2H, d, J = 8.4 Hz, H_{24}), 7.24 (1H, d, J = 8.6 Hz, H_{20}), 7.22 (1H, s, H_{13}), 6.96 (1H, d, J = 8.3 Hz, H_{12}), 4.94 (1H, s, br, H_{15}), 4.25 (2H, d, J = 5.2 Hz, H_{14}), 3.96 (3H, t, J = 7.2 Hz, H_{10}), 1.72–1.67 (2H, m, H_9), 1.43 (9H, s, H_{18}), 1.38–1.32 (2H, m, H_8), 1.31–1.21 (12H, m, H_7–H_2), 0.88 (3H, t, J = 8.1 Hz, H_1); **^{13}C NMR** (150 MHz, CD_2Cl_2) δ: 155.1, 148.5, 139.1, 131.6, 131.4, 129.8, 128.6, 128.3, 123.9, 120.6, 118.8 (q, J = 320 Hz), 112.5, 79.1, 68.6, 43.9, 31.9, 29.6, 29.5, 29.3, 29.2, 29.1, 28.1, 26.1, 22.7, 13.9; **^{19}F NMR** (376 MHz, $CDCl_3$): -72.83;

MS (ESI$^+$): **m/z** = 588.6 [100, $(M+H)^+$], 610.7 [15, $(M+Na)^+$]; **HRMS** (ESI$^+$): $[C_{29}H_{44}N_2O_6S]^+$ predicted 605.2867, found 605.2861 (Δ − 0.9 ppm).

3. *tert*-Butyl ((6-(decyloxy)-4'-(4,4,5,5-tetramethyl-1,3,2-dioxaborolan-2-yl)-[1,1'-biphenyl]-3-yl)methyl)carbamate

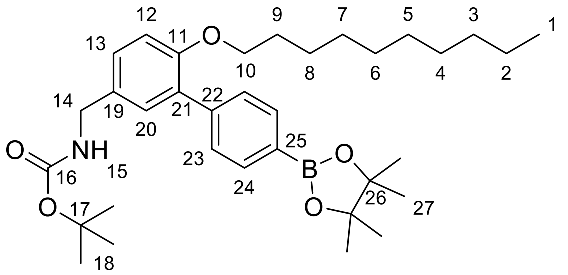

To a stirred solution of **S3** (270 mg, 0.459 mmol) in dioxane (12 ml) were added KOAc (200 mg, 2.02 mmol) and bis(pinocolato)diboron (270 mg, 1.06 mmol). The suspension was sparged with argon for 15 min, then {[1,1'-Bis(diphenylphosphino)ferrocene] dichloropalladium(II), complex with CH_2Cl_2} (60 mg, 7.35 µmol) was added. The reaction was heated to 100 °C for 16 hours under argon, then cooled to r.t. and concentrated *in vacuo*. The residue was purified by flash column chromatography [on Al_2O_3 (Brockmann II), EtOAc:PE 1:99 to 1:2] to furnish **3** (209 mg, 0.370 mmol, 81%) as a colorless oil.

^{1}H NMR (600 MHz, $CDCl_3$) *δ*: 7.83 (2H, d, *J* = 8.3 Hz, H_{23}), 7.55 (2H, d, *J* = 8.1 Hz, H_{24}), 7.24 (1H, d, *J* = 1.9 Hz, H_{13}), 7.21 (1H, d, *J* = 8.6 Hz, H_{20}), 6.92 (1H, d, *J* = 8.6 Hz, H_{12}), 4.78 (1H, s, br, H_{15}), 4.29 (2H, d, *J* = 6.3 Hz, H_{14}), 3.92 (3H, t, *J* = 8.4 Hz, H_{10}), 1.72–1.65 (2H, m, H_9), 1.46 (9H, s, H_{18}), 1.39–1.34 (14H, m, H_8 + H_{27}), 1.31–1.21 (12H, m, H_7–H_2), 0.88 (3H, t, *J* = 8.1 Hz, H_1); **^{13}C NMR** (150 MHz, $CDCl_3$) *δ*: 155.9, 155.4, 141.3, 134.3, 131.0, 130.8, 130.2, 128.9, 128.0, 112.7, 83.7, 79.5, 68.7, 44.2, 31.9, 29.6, 29.6, 29.3, 29.3, 29.2, 29.1, 28.5, 26.0, 24.9, 22.7, 14.1; **MS** (ESI$^+$): **m/z** = 565.4 [100, $(M+H)^+$], 588.3 [35, $(M+Na)^+$]; **HRMS** (ESI$^+$): $[C_{34}H_{56}N_2O_5B]^+$ predicted 582.4313, found 582.4306 (Δ − 1.2 ppm).

S4. *tert*-Butyl ((6-(decyloxy)-4'-(6-(dimethoxymethyl)pyridin-2-yl)-[1,1'-biphenyl]-3-yl)methyl)carbamate

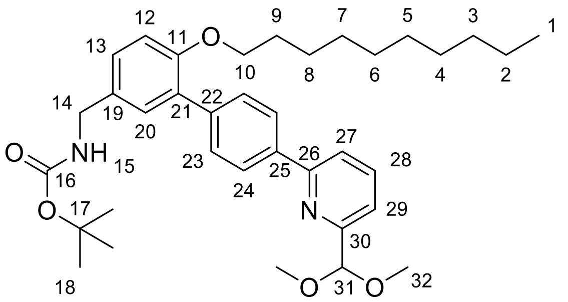

To a stirred solution of **3** (170 mg, 0.301 mmol) in THF (5 ml) and 2 M Na_2CO_3 $_{(aq)}$ (2 ml) was added 2-Bromo-6-(dimethoxymethyl)pyridine (**7**) (160 mg, 0.689 mmol). The solution was sparged with argon for 15 min. $Pd(PPh_3)_4$ (120 mg, 0.104 mmol) was added, and the reaction heated to 60 °C under argon for 4 days. The reaction was cooled to r.t. and partitioned between EtOAc (50 ml) and 0.1 M $EDTA_{(aq)}$ (50 ml). The organic phase was dried over $MgSO_4$, filtered and concentrated *in vacuo*. The crude residue was purified by flash column chromatography [EtOAc:CH_2Cl_2 1:99 grading to 1:24] to furnish **S4** (162 mg, 0.274 mmol, 91%) as a pale yellow oil.

^{1}H NMR (600 MHz, $CDCl_3$) δ: 8.06 (2H, d, J = 8.1 Hz, H_{23}), 7.83–7.80 (1H, m, H_{28}), 7.75 (1H, d, J = 8.2 Hz, H_{29}), 7.65 (2H, d, J = 7.8 Hz, H_{24}), 7.51 (1H, d, J = 7.8 Hz, H_{27}), 7.28 (1H, d, J = 2.6 Hz, H_{20}), 7.23 (1H, d, J = 8.4 Hz, H_{13}), 6.94 (1H, d, J = 8.4 Hz, H_{12}), 5.48 (1H, s, H_{31}), 4.81 (1H, s, br, H_{15}), 4.31 (2H, d, J = 5.4 Hz, H_{14}), 3.95 (3H, t, J = 6.9 Hz, H_{10}), 1.74–1.68 (2H, m, H_9), 1.46 (9H, s, H_{18}), 1.41–1.35 (2H, m, H_8), 1.31–1.19 (12H, m, H_7–H_2), 0.86 (3H, t, J = 7.2 Hz, H_1); **^{13}C NMR** (150 MHz, $CDCl_3$) δ: 157.3, 156.5, 155.9, 155.5, 139.2, 137.5, 134.5, 131.1, 130.5, 130.1, 129.9, 128.0, 126.6, 120.3, 119.3, 112.7, 104.9, 79.5, 68.7, 54.2, 44.2, 31.9, 29.6, 29.6, 29.3, 29.3, 29.2, 28.4, 28.1, 22.7, 14.1; **MS** (ESI^+): **m/z =** 591.4 [100, $(M+H)^+$], 613.4 [15, $(M+Na)^+$]; **HRMS** (ESI^+): $[C_{36}H_{51}N_2O_5]^+$ predicted 591.3792, found 591.3779 (Δ – 2.3 ppm).

9. *tert*-Butyl ((6-(decyloxy)-4'-(6-formylpyridin-2-yl)-[1,1'-biphenyl]-3-yl)methyl)carbamate

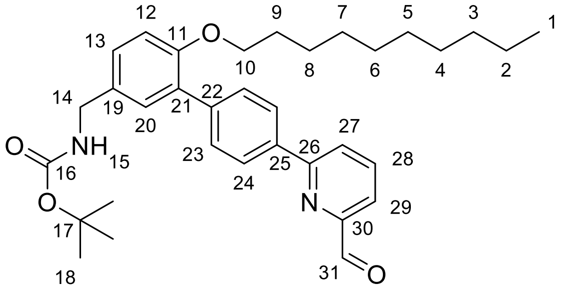

To a stirred solution of **S4** (78.0 mg, 0.132 mmol) in THF (3 ml) was added H_2O (100 µl) and *p*-toluenesulfonic acid (78.0 mg, 0.411 mmol). The reaction was heated under argon to 50 °C for 3 h. The reaction was partitioned between CH_2Cl_2 (30 ml) and 2 M $NaOH_{(aq)}$ (30 ml). The organic phase was dried over Na_2SO_4, filtered and concentrated *in vacuo*. The crude residue was purified by flash column chromatography [EtOAc:CH_2Cl_2 1:99 grading to 1:24] to furnish **9** (72 mg, 0.132 mmol, quant.) as a pale yellow oil.

^{1}H NMR (600 MHz, $CDCl_3$) δ: 10.20 (1H, s, H_{31}), 8.13 (2H, d, J = 8.1 Hz, H_{23}), 8.01 (1H, dd, J = 7.6, 1.1 Hz, H_{29}), 7.95 (1H, t, J = 7.6 Hz, H_{28}), 7.91 (1H, dd, J = 7.6, 1.1 Hz, H_{27}), 7.70 (2H, d, J = 8.4 Hz, H_{24}), 7.30 (1H, d, J = 2.2 Hz, H_{20}), 7.26–7.22 (1H, d, J = 8.6 Hz, H_{13}), 6.95 (1H, d, J = 8.4 Hz, H_{12}), 4.82 (1H, s, br, H_{15}), 4.31 (2H, d, J = 5.2 Hz, H_{14}), 3.97 (3H, t, J = 6.2 Hz, H_{10}), 1.75–1.69 (2H, m, H_9), 1.47 (9H, s, H_{18}), 1.42–1.35 (2H, m, H_8), 1.31–1.18 (12H, m, H_7–H_2), 0.85 (3H, t, J = 6.9 Hz, H_1); **^{13}C NMR** (150 MHz, $CDCl_3$) δ: 194.1, 157.8, 155.9, 155.5, 152.7, 139.9, 137.8, 136.5, 131.1, 130.2, 130.2, 130.2, 128.2, 126.5, 124.4, 119.7, 112.7, 79.5, 68.7, 44.2, 38.1, 31.9, 29.6, 29.5, 29.3, 29.3, 29.2, 28.4, 26.1, 22.7, 14.1; **MS** (ESI^+): **m/z =** 545.3 [100, $(M+H)^+$], 567.2 [45, $(M+Na)^+$]; **HRMS** (ESI^+): $[C_{34}H_{45}N_2O_4]^+$ predicted 545.3374, found 545.3374 (Δ – 1.4 ppm).

11. ***tert*-Butyl (*E*)-((6-(decyloxy)-4'-(6-((2-(4-nitrophenyl)hydrazono)methyl)pyridin-2-yl)-[1,1'-biphenyl]-3-yl)methyl)carbamate**

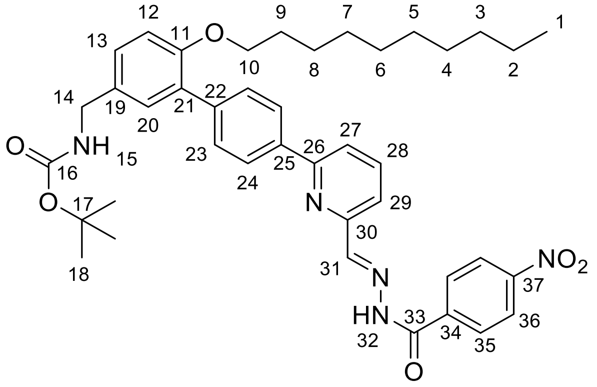

To a stirred solution of **9** (740 mg, 1.36 mmol) in CH_2Cl_2 (100 ml) was added 4-nitrobenzohydrazide (296 mg, 1.63 mmol, **21**) and aniline (6.33 mg, 0.0680 mmol). The reaction was stirred for 16 h then concentrated *in vacuo*. The crude residue was purified by flash column chromatography [MeOH:CH_2Cl_2 1:19 grading to 1:9] to furnish **11** (800 mg, 1.13 mmol, 83%) as a yellow oil.

^{1}H NMR (600 MHz, DMSO-d_6) δ: 15.39 (1H, s, H_{32}), 8.24–8.19 (3H, m, $H_{29} + H_{35}$), 8.09 (1H, d, J = 7.4 Hz, H_{27}), 8.06 (2H, d, J = 9.5 Hz, H_{34}), 7.89–7.84 (4H, m, $H_{24} + H_{28} + H_{31}$), 7.54 (2H, d, J = 7.4 Hz, H_{23}), 7.26–7.21 (2H, m, $H_{13} + H_{20}$), 7.09 (1H, d, J = 7.4 Hz, H_{12}), 4.16 (2H, d, J = 5.0 Hz, H_{14}), 3.94 (2H, t, J = 5.0 Hz, H_{10}), 1.55–1.49 (2H, m, H_9), 1.39 (9H, s, H_{18}), 1.20–1.17 (2H, m, H_8), 1.12–1.06 (4H, m, $H_7 + H_6$), 1.04–0.98 (8H, m, H_5–H_2), 0.83 (3H, t, J = 6.6 Hz, H_1); **^{13}C NMR** (150 MHz, DMSO-d_6) δ: 162.7, 156.5, 156.2, 155.0, 152.3, 149.8, 140.9, 140.3, 140.2, 139.5, 136.7, 132.9, 130.2, 129.7, 129.5, 129.1, 128.3, 127.3, 126.1, 124.4, 123.1, 113.4, 68.7, 41.8, 31.6, 29.4, 29.2, 29.2, 29.0, 29.0, 28.7, 28.7, 26.0, 22.5, 14.3; **MS** (ESI$^+$): **m/z =** 708.1 [100, $(M+H)^+$], 730.1 [15, $(M+Na)^+$]; **HRMS** (ESI$^+$): $[C_{41}H_{49}N_5O_6]^+$ predicted 708.3756, found 708.3751 (Δ −0.7 ppm).

1. ***N'*-((*E*)-(6-(2'-(Decyloxy)-5'-(((2-(((*S*)-((1*S*,2*R*,4*S*,5*R*)-5-ethylquinuclidin-2-yl)(6-methoxyquinolin-4-yl)methyl)amino)-3,4-dioxocyclobutyl)amino)methyl)-[1,1'-biphenyl]-4-yl)pyridin-2-yl)methylene)-4-nitrobenzohydrazide**

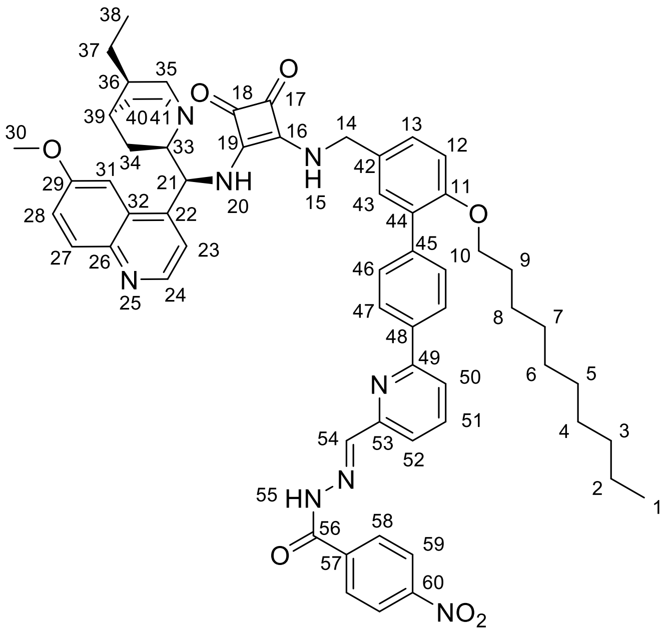

Hydrazide **11** (51.1 mg, 0.0721 mmol) was dissolved in CH_2Cl_2 (4 ml) and TFA (1 ml) was added. The reaction was stirred at r.t. for 1 h and concentrated *in vacuo* with the addition of toluene (5 ml). The residue was azeotroped 5 × with toluene:CH_2Cl_2 (1:1, 10 ml). The crude residue was dissolved in DMF (2 ml), MeOH (2 ml) and Et_3N (0.5 ml), then **4** (85.2 mg, 0.195 mmol) was added. The reaction was stirred at r.t. for 16 h, concentrated *in vacuo*, and purified by preparatory thin layer chromatography (4 × 500 μm, eluted with Et_3N:MeOH:CH_2Cl_2 0.1:1:10) to afford **1** (46 mg, 0.0455 mmol, 63%) as a yellow powder.

^{1}H NMR (600 MHz, DMSO-d_6) δ: 12.40 (1H, s, H_{55}), 8.75 (1H, s, H_{24}), 8.61 (1H, s, H_{54}), 8.41 (2H, d, J = 9.3 Hz, H_{59}), 8.20 (2H, d, J = 9.3 Hz, H_{58}), 8.16–8.10 (1H, m, H_{51}), 8.06–7.93 (5H, m, H_{27} + H_{47} + H_{50} + H_{52}), 7.63–7.54 (3H, m, H_{23} + H_{46}), 7.42 (1H, dd, J = 9.3, 2.3 Hz, H_{13}), 7.34–7.31 (2H, m, H_{28} + H_{31}), 7.29–7.23 (1H, m, H_{43}), 7.11–7.05 (1H, m, H_{12}), 5.94 (2H, br, H_{15} + H_{20}), 4.74–4.70 (2H, m, H_{14}), 3.99–3.87 (5H, m, H_{10} + H_{30}), 3.15–3.07 (1H, m, H_{21}), 2.42–2.35 (1H, m, H_{33}), 1.65–1.58 (2H, m, H_9), 1.56–1.44 (3H, m, H_8 + H_{35a}), 1.12–1.06 (4H, m, H_7 + H_6), 1.42–1.10 (23H, m, H_2–H_7 + H_{34} + H_{35b} + H_{36} + H_{37} + H_{39} + H_{40} + H_{41}), 0.81–0.74 (6H, m, H_1 + H_{38}); **^{13}C NMR** (150 MHz, DMSO-d_6) δ: 182.8, 182.5, 162.3, 156.2, 155.6, 153.4, 149.9, 149.8, 148.2, 144.8, 140.9, 140.2, 139.4, 139.3, 138.5, 137.0, 132.0, 131.3, 130.5, 130.0, 129.8, 129.5, 129.4, 129.2, 128.7, 127.3, 126.6, 125.8, 124.2, 123.4, 121.4, 119.3, 113.6, 101.9, 68.6, 59.1, 56.2, 48.6, 40.7, 31.7, 31.6, 29.4, 29.3, 29.2, 29.2, 29.1, 29.1, 29.0, 29.0, 28.9, 25.9, 25.9, 22.5, 22.5, 14.4, 14.3; **MS** (ESI$^+$): **m/z =** 1011.1 [100, $(M+H)^+$], 1033.2 [5, $(M+Na)^+$]; **HRMS** (ESI$^+$): $[C_{60}H_{67}N_8O_7]^+$ predicted 1011.5127, found 1011.5120 (Δ − 0.7 ppm).

S5. *tert*-Butyl ((6-(decyloxy)-4'-(2-(dimethoxymethyl)pyridin-3-yl)-[1,1'-biphenyl]-3-yl)methyl)carbamate

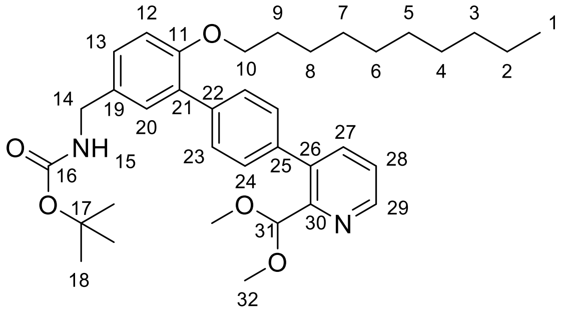

To a stirred solution of **3** (170 mg, 0.301 mmol) in THF (5 ml) and 2 M $Na_2CO_{3(aq)}$ (2 ml) was added **6** (160 mg, 0.689 mmol). The solution was sparged with argon for 15 min, $Pd(PPh_3)_4$ (120 mg, 0.104 mmol) was added, and the reaction heated to 60 °C under argon for 4 days. The reaction was cooled to r.t. and partitioned between EtOAc (50 ml) and 0.1 M $EDTA_{(aq)}$ (50 ml). The organic phase was dried over $MgSO_4$, filtered and concentrated *in vacuo*. The crude residue was purified by flash column chromatography [EtOAc:CH_2Cl_2 1:99 grading to 1:24] to furnish **S5** (162 mg, 0.274 mmol, 91%) as a pale yellow oil.

^{1}H NMR (600 MHz, $CDCl_3$) δ: 8.08 (2H, d, J = 8.4 Hz, H_{23}), 7.84–7.81 (1H, m, H_{28}), 7.76 (1H, d, J = 7.7 Hz, H_{29}), 7.69 (2H, d, J = 8.4 Hz, H_{24}), 7.52 (1H, d, J = 7.7 Hz, H_{27}), 7.30 (1H, d, J = 2.1 Hz, H_{20}), 7.25 (1H, d, J = 8.4 Hz, H_{13}), 6.96 (1H, d, J = 8.4 Hz, H_{12}), 5.47 (1H, s, H_{31}), 4.83 (1H, s, br, H_{15}), 4.33 (2H, d, J = 5.8 Hz, H_{14}), 3.98 (2H, t, J = 6.7 Hz, H_{10}), 3.50 (6H, s, H_{32}), 1.76–1.71 (2H, m, H_9), 1.49 (9H, s, H_{18}), 1.44–1.38 (2H, m, H_8), 1.34–1.21 (12H, m, H_7–H_2), 0.88 (3H, t, J = 7.2 Hz, H_1); **^{13}C NMR** (150 MHz, $CDCl_3$) δ: 157.3, 156.6, 155.9, 155.5, 139.1, 137.6, 137.3,

131.0, 130.5, 130.1, 129.9, 128.0, 126.6, 120.2, 119.2, 112.7, 105.0, 68.8, 54.1, 44.2, 31.9, 29.6, 29.6, 29.5, 29.3, 29.3, 29.1, 28.4, 26.1, 22.7, 14.1; **MS** (ESI$^+$): **m/z =** 591.4 [100, $(M+H)^+$], 613.3 [20, $(M+Na)^+$]; **HRMS** (ESI$^+$): $[C_{36}H_{50}N_2O_5]^+$ predicted 591.3792, found 591.3779 (Δ − 2.3 ppm).

8. *tert*-Butyl ((6-(decyloxy)-4'-(2-formylpyridin-3-yl)-[1,1'-biphenyl]-3-yl)methyl)carbamate

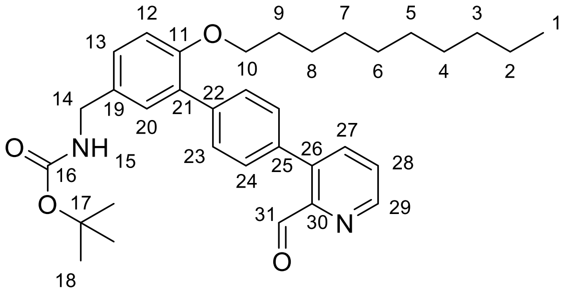

To a stirred solution of **S5** (60.0 mg, 0.102 mmol) in THF (3 ml) was added H_2O (100 µl) and *p*-toluenesulfonic acid (60.0 mg, 0.316 mmol). The reaction was heated under argon to 50 °C for 3 h. The reaction was partitioned between CH_2Cl_2 (30 ml) and 2 M $NaOH_{(aq)}$ (30 ml). The organic phase was dried over Na_2SO_4, filtered and concentrated *in vacuo*. The crude residue was purified by flash column chromatography [EtOAc:CH_2Cl_2 1:99 grading to 1:24] to furnish **8** (44.7 mg, 0.0822 mmol, 81%) as a pale yellow oil.

^{1}H NMR (600 MHz, Acetone-*d$_6$*) *δ*: 10.14 (1H, s, H_{31}), 8.32–8.26 (3H, m, H_{23} + H_{29}), 8.15–8.11 (1H, m, H_{28}), 7.90 (1H, d, *J* = 7.7 Hz, H_{27}), 7.74 (2H, d, *J* = 7.7 Hz, H_{24}), 7.39 (1H, d, *J* = 2.2 Hz, H_{20}), 7.29 (2H, dd, *J* = 7.8, 2.2 Hz, H_{13}), 7.07 (1H, d, *J* = 8.4 Hz, H_{12}), 6.47 (1H, s, br, H_{15}), 4.28 (2H, d, *J* = 6.4 Hz, H_{14}), 4.04 (2H, t, *J* = 6.8 Hz, H_{10}), 1.76–1.70 (2H, m, H_9), 1.46–1.40 (11H, m, H_{18} + H_8), 1.34–1.16 (12H, m, H_7–H_2), 0.83 (3H, t, *J* = 6.3 Hz, H_1); **^{13}C NMR** (150 MHz, Acetone-*d$_6$*) *δ*: 193.4, 157.2, 155.9, 155.2, 152.9, 140.3, 138.4, 136.3, 132.7, 130.0, 129.7, 129.6, 128.1, 126.3, 124.3, 119.4, 112.7, 77.8, 68.3, 43.4, 31.7, 29.5, 29.2, 29.2, 29.0, 29.0, 28.8, 27.8, 26.0, 22.4, 13.5; **MS** (ESI$^+$): **m/z =** 545.3 [100, $(M+H)^+$], 567.3 [10, $(M+Na)^+$]; **HRMS** (ESI$^+$): $[C_{34}H_{45}N_2O_4]^+$ predicted 545.3374, found 545.3366 (Δ − 1.4 ppm).

10. *tert*-Butyl (*E*)-((6-(decyloxy)-4'-(2-((2-(3-nitrobenzoyl)hydrazono)methyl)pyridin-3-yl)-[1,1'-biphenyl]-3-yl)methyl)carbamate

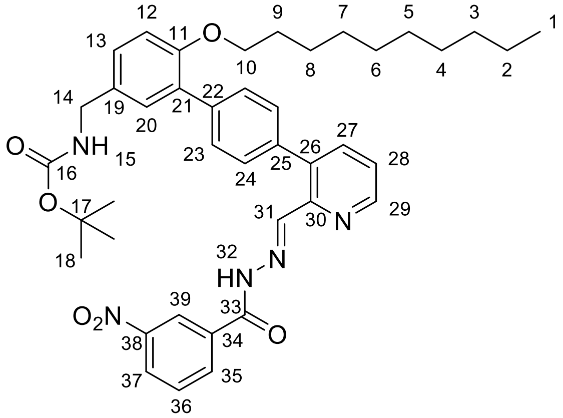

To a stirred solution of **8** (42.0 mg, 0.0773 mmol) in CH_2Cl_2 (100 ml) was added 3-nitrobenzohydrazide (18.0 mg, 0.0928 mmol, **22**) and aniline (0.360 mg, 3.87 µmol). The reaction was stirred for 16 h then concentrated *in vacuo*. The crude residue was purified by flash column

chromatography [MeOH:CH_2Cl_2 1:19 grading to 1:9] to furnish **10** (43.7 mg, 0.617 mmol, 80%) as a yellow oil.

^{1}H NMR (600 MHz, $CDCl_3$) δ: 15.82 (1H, s, H_{32}), 8.75 (1H, s, H_{39}), 8.36 (1H, d, J = 8.2 Hz, H_{37}), 8.13 (1H, d, J = 8.2 Hz, H_{35}), 8.05–8.01 (1H, m, H_{28}), 7.81 (1H, d, J = 8.2 Hz, H_{29}), 7.74–7.70 (3H, m, H_{23} + H_{20}), 7.54–7.49 (3H, m, H_{24} + H_{27}), 7.47–7.43 (1H, m, H_{36}), 7.28 (1H, d, J = 8.5 Hz, H_{13}), 6.96 (1H, d, J = 8.5 Hz, H_{12}), 4.96 (1H, s, br, H_{15}), 4.35 (2H, d, J = 4.8 Hz, H_{14}), 3.98 (2H, t, J = 6.7 Hz, H_{10}), 1.72–1.65 (2H, m, H_9), 1.47 (9H, s, H_{18}), 1.36–1.30 (2H, m, H_8), 1.27–1.14 (12H, m, H_7–H_2), 0.83 (3H, t, J = 6.6 Hz, H_1); **^{13}C NMR** (150 MHz, $CDCl_3$) δ: 162.9, 157.6, 156.0, 155.4, 152.2, 148.3, 140.5, 140.0, 138.9, 136.7, 135.1, 133.4, 131.3, 130.2, 130.1, 129.9, 129.7, 128.5, 126.9, 126.6, 124.6, 123.5, 122.5, 112.7, 68.7, 44.2, 31.9, 29.6, 29.5, 29.5, 29.3, 29.3, 29.2, 29.1, 26.0, 22.7, 14.1; **MS** (ESI^+): **m/z =** 708.1 [100, $(M+H)^+$], 730.1 [10, $(M+Na)^+$]; **HRMS** (ESI^+): $[C_{41}H_{49}N_5O_6Na]^+$ predicted 730.3575, found 730.3581 (Δ + 0.8 ppm).

2. *N'*-((*E*)-(3-(2'-(decyloxy)-5'-(((2-(((*R*)-((1*S*,2*S*,4*S*,5*R*)-5-ethylquinuclidin-2-yl)(6-methoxyquinolin-4-yl)methyl)amino)-3,4-dioxocyclobutyl)amino)methyl)-[1,1'-biphenyl]-4-yl)pyridin-2-yl)methylene)-3-nitrobenzohydrazide

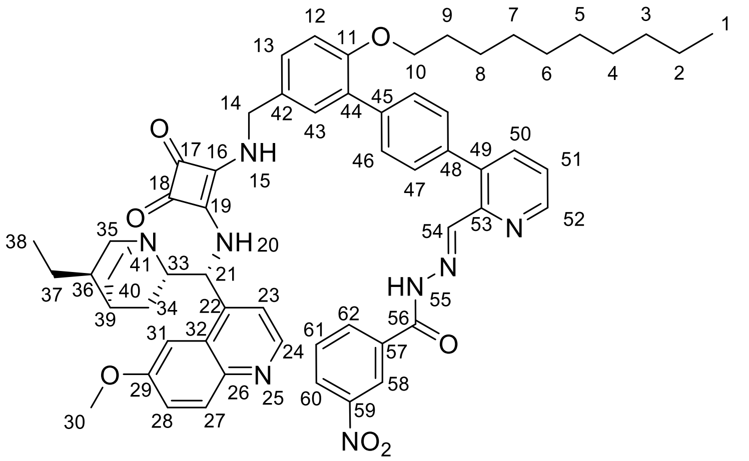

10 (43.7 mg, 0.617 mmol) was dissolved in CH_2Cl_2 (4 ml) and TFA (1 ml) was added. The reaction was stirred at r.t. for 1 h and concentrated *in vacuo* with the addition of toluene (5 ml). The residue was azeotroped 5 × with toluene:CH_2Cl_2 (1:1, 10 ml). The crude residue was dissolved in DMF (2 ml), MeOH (2 ml) and Et_3N (0.5 ml), then **5** (85 mg, 0.195 mmol) was added. The reaction was stirred at r.t. for 16 h, concentrated *in vacuo*, and purified by preparatory thin layer chromatography (4 × 500 µm, eluted with Et_3N:MeOH:CH_2Cl_2 0.1:1:10) to afford **2** (33.1 mg, 0.327 mmol, 53%) as a yellow powder.

^{1}H NMR (600 MHz, DMSO-d_6) δ: 12.42 (1H, s, H_{55}), 8.80 (1H, s, H_{55}), 8.75 (1H, s, H_{24}), 8.63 (1H, s, H_{54}), 8.49 (1H, d, J = 8.1 Hz, H_{60}), 8.41 (1H, d, J = 8.1 Hz, H_{62}), 8.15 (2H, d, J = 8.1 Hz, H_{47}), 8.07 (1H, d, J = 6.9 Hz, H_{50}), 8.04–7.94 (3H, m, H_{51} + H_{52} + H_{61}), 7.91–7.87 (1H, m, H_{27}), 7.86–7.79 (1H, m, H_{23}), 7.65–7.55 (3H, m, H_{13} + H_{46}), 7.43 (1H, d, J = 8.1, H_{28}), 7.36-7.33 (1H, m, H_{31}), 7.29–7.25 (1H, m, H_{43}), 7.11–7.07 (1H, m, H_{12}), 6.29 (1H, br, H_{15} *or* H_{20}), 6.05 (1H, br, H_{15} *or* H_{20}), 4.70–4.61 (2H, m, H_{14}), 3.99–3.95 (2H, m, H_{10}), 3.94–3.87 (4H, m, H_{21} + H_{30}), 2.90–2.84 (1H, m, H_{33}), 1.65–1.59 (2H, m, H_9), 1.56–1.45 (5H, m, H_7 + H_8 + H_{35a}), 1.38–1.10 (20 H, m, H_2–H_6 + H_{34} + H_{35b} + H_{36} + H_{37} + H_{39} + H_{40} + H_{41}), 0.84–0.80 (3H, m, H_1), 0.77 (3H, t, J = 7.4, H_{38}); **^{13}C NMR** (150 MHz, DMSO-d_6) δ: 182.4, 182.1, 167.0, 157.9, 155.1, 147.8,

147.7, 146.7, 145.2, 144.3, 141.2, 139.6, 138.5, 137.9, 137.3, 134.2, 133.8, 131.6, 131.1, 130.2, 129.9, 129.7, 129.6, 129.4, 129.3, 128.9, 127.5, 126.0, 125.6, 125.2, 122.5, 122.1, 113.2, 101.3, 68.2, 59.0, 55.6, 49.0, 48.1, 46.4, 31.3, 29.1, 29.0, 28.9, 28.9, 28.8, 28.7, 28.7, 28.6, 28.6, 25.7, 25.6, 25.4, 22.1, 13.9, 13.9; **MS** (ESI^+): **m/z =** 1011.1 [100, $(M+H)^+$], 1033.2 [5, $(M+Na)^+$]; **HRMS** (ESI^+): $[C_{60}H_{67}N_8O_7]^+$ predicted 1011.5127, found 1011.5096 (Δ − 3.1 ppm).

3. Catalysis tests

Addition of malonitrile to chalcone to form S6

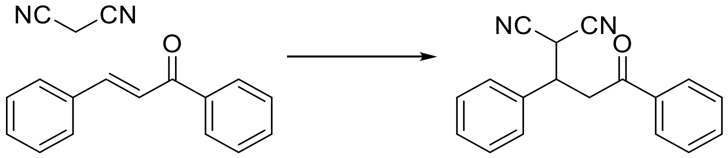

Malonitrile **12** (1.0 mg, 0.015 mmol), chalcone **S7** (32 mg, 0.15 mmol) and catalyst **1** or **2** (0.75 mg, 0.75 µmol) were dissolved in $CDCl_3$ (0.7 ml). Conversion was measured periodically by ^1H NMR spectroscopy and comparison with authentic spectra of starting materials and product. Upon completion, the crude was purified by TLC, and the chirality of the product ascertained by HPLC (chiralpak IC, 90:10 Hexane:*i*PrOH, major enantiomer produced by catalyst **1** t_r = 18.4 min, for catalyst **2** t_r = 29.9 min).

Addition of 1,4-dithiane-2,5-diol to chalcone to form S8

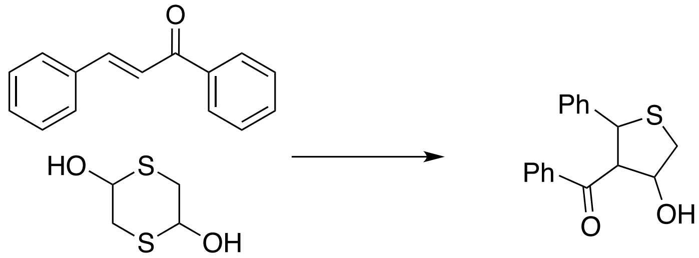

1,4-dithiane-2,5-diol **S9** (2.3 mg, 0.015 mmol), chalcone **S7** (32 mg, 0.15 mmol) and catalyst **1** or **2** (0.75 mg, 0.75 µmol) were dissolved in toluene (0.7 ml) then heated to 50 °C. Conversion was measured periodically by ^1H NMR spectroscopy and comparison with authentic spectra of starting materials and product. Upon completion, the crude was purified by TLC, and the chirality of the product ascertained by HPLC (chiralpak IA, 90:10 Hexane:*i*PrOH, major enantiomer produced by catalyst **1** t_r = 13.6 min, for catalyst **2** t_r = 19.3 min).

Addition of malonitrile to (*E*)-1-(furan-2-yl)-3-phenylprop-2-en-1-one to form 14

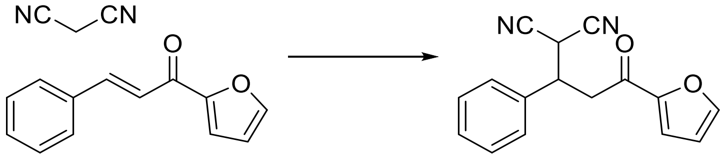

Malonitrile **12** (1.0 mg, 0.015 mmol), (*E*)-1-(furan-2-yl)-3-phenylprop-2-en-1-one **13** (30 mg, 0.15 mmol) and catalyst **1** or **2** (0.75 mg, 0.75 µmol) were dissolved in $CDCl_3$ (0.7 ml). Conversion was measured periodically by ^1H NMR spectroscopy and comparison with authentic spectra of starting materials and product. Upon completion, the crude was purified by TLC, and the chirality of the product ascertained by HPLC (chiralpak IA, 90:10 Hexane:*i*PrOH, major enantiomer produced by catalyst **1** t_r = 19.9 min, for catalyst **2** t_r = 23.9 min).

Addition of diethyl malonate to *trans*-β-nitrostyrene to form S10

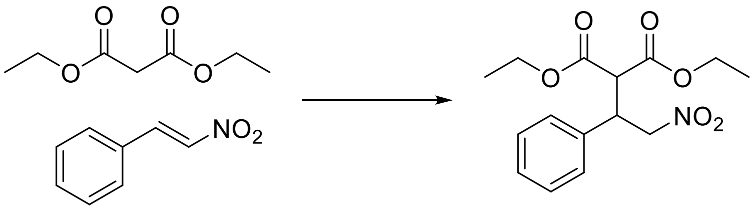

Diethyl malonate **S11** (24 mg, 0.15 mmol), *trans*-β-nitrostyrene **S12** (2.2 mg, 0.015 mmol) and catalyst **1** or **2** (3.75 mg, 3.75 μmol) were dissolved in $CDCl_3$ (0.7 ml). Conversion was measured periodically by ^1H NMR spectroscopy and comparison with authentic spectra of starting materials and product. Upon completion, the crude was purified by TLC, and the chirality of the product ascertained by HPLC (chiralpak IA, 90:10 Hexane:*i*PrOH, major enantiomer produced by catalyst **1** t_r = 13.7 min, for catalyst **2** t_r = 18.4 min).

Addition of diethyl malonate to *trans*-β-nitrostyrene to form S13

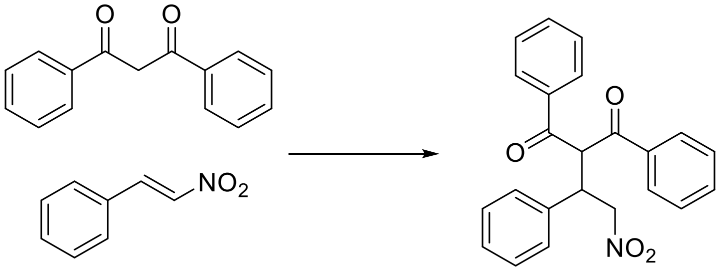

1,3-Diphenylpropane-1,3-dione **S14** (3.4 mg, 0.015 mmol), *trans*-β-nitrostyrene **S12** (22 mg, 0.15 mmol) and catalyst **1** or **2** (3.75 mg, 3.75 μmol) were dissolved in $CDCl_3$ (0.7 ml). Conversion was measured periodically by ^1H NMR spectroscopy and comparison with authentic spectra of starting materials and product. Upon completion, the crude was purified by TLC, and the chirality of the product ascertained by HPLC (chiralpak IA, 90:10 Hexane:*i*PrOH, major enantiomer produced by catalyst **1** t_r = 18.5 min, for catalyst **2** t_r = 36.8 min).

Addition of malonitrile to (*E*)-1-(furan-2-yl)-3-phenylprop-2-en-1-one to form S15

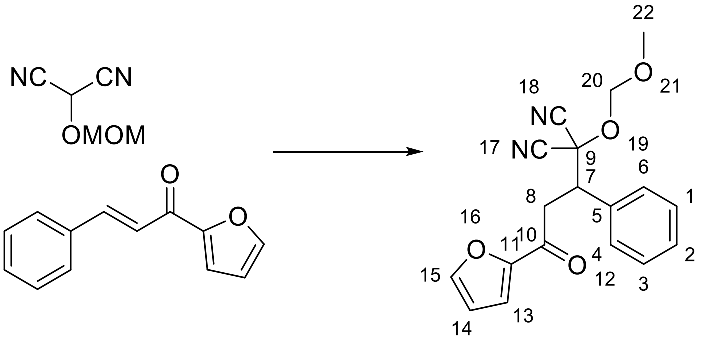

2-(Methoxymethoxy)malononitrile **S16** (8.0 mg, 0.063 mmol, prepared according to literature procedure)[S10], (*E*)-1-(furan-2-yl)-3-phenylprop-2-en-1-one **13** (62 mg, 0.32 mmol) and catalyst **1** or **2** (3.2 mg, 3.2 μmol) were dissolved in $CDCl_3$ (1 ml). Upon completion, the crude was purified by PTLC, furnishing a colourless oil (24.1 mg, 0.0744 mmol, 74%) and the chirality of the product ascertained by HPLC (chiralpak IA, 90:10 Hexane:*i*PrOH, major enantiomer produced by catalyst **1** t_r = 12.1 min, for catalyst **2** t_r = 9.8 min). **^{1}H NMR** (600 MHz, $CDCl_3$) *δ*: 7.60 (1H, d, *J* = 1.9 Hz, H_{15}), 7.49 (2H, dd, *J* = 7.8, 1.3 Hz, $H_4 + H_6$), 7.38-7.34 (3H, m, H_1–H_3), 7.21 (1H, d, *J* = 3.6 Hz, H_{13}), 6.54

(1H, dd, *J* = 3.6, 1.7 Hz, H_{14}), 5.06–5.01 (2H, m, H_{20}), 4.21 (1H, dd, *J* = 9.2, 3.9 Hz, H_7), 3.72 (1H, dd, *J* = 15.8, 9.7 Hz, H_{8a}), 3.55 (1H, dd, *J* = 16.7, 3.9 Hz, H_{8b}), 3.46 (3H, s, H_{22}); **^{13}C NMR** (150 MHz, $CDCl_3$) δ: 184.4, 152.2, 146.8, 133.9, 129.6, 129.3, 128.9, 117.6, 112.7, 96.5, 70.0, 57.5, 49.1, 38.4; **MS** (ESI^+): **m/z =** 347.1 [100, $(M+Na)^+$]; **HRMS** (ESI^+): $[C_{18}H_{16}N_2O_4Na]^+$ predicted 347.1002, found 347.1002 (Δ −0.1 ppm).

4. Optimisation of light switching conditions for catalyst 1

The degree of isomerisation was monitored by following the integral ratio of the *E* and *Z* states of hydrazone proton H(55) (at c. 12 ppm and c. 16 ppm, see characterisation for full numbering).

A brief initial screen of light sources showed a 395 nm source to be the most efficient in promoting hydrazone isomerization. Initially problems with decomposition, particularly hydrazone hydrolysis, were observed. This could be prevented by the addition of molecular sieves, which also improved the *E*:*Z* ratio achieved. A significant improvement in final switching ratio was achieved by the addition of EtOAc to the solvent mixture (pure EtOAc could not be used due to insufficient solubility). Increasing the concentration of solution was deleterious to conversion. The timescale of switching could be accelerated to 20 min of irradiation by increasing the power and using CH_2Cl_2:EtOAc as solvent. This switching system also alleviated the previously observed decomposition, obviating the need for molecular sieves.

Table S1

Mass/ mg	Solvent	Additive	Wavelength/nm	Time/min	Power / mW	Ratio *E:Z*	Decomp.
1	CH_2Cl_2	-	365	30	215	60:40	**20%**
1	Toluene	-	365	30	215	57:43	**20%**
1	EtOAc	-	365	30	215	43:57	**20%**
1	$CHCl_3$	-	395	30	215	58:42	**10%**
1	Toluene: CH_2Cl_2 9:1	-	395	30	215	60:40	**10%**
1	EtOAc: CH_2Cl_2 9:1	-	395	30	215	40:60	**10%**
1	CH_2Cl_2	-	Cold White	30	215	87:13	**10%**
1	EtOAc: CH_2Cl_2 9:1	-	Cold White	30	215	90:10	**10%**
1	EtOAc CH_2Cl_2 9:1	-	Cold White	900	215	45:55	**25%**
1	EtOAc: $CHCl_3$ 4:1	-	395	300	380	54:46	**35%**
**1**	**$CHCl_3$**	**MS**	**395**	**60**	**380**	**40:60**	**-**
1	$CHCl_3$	MS	395	140	380	46:54	5%
1	EtOAc: $CHCl_3$ 4:1	MS, 10 Eq Chalcone	395	60	380	29:71	-
1	EtOAc: $CHCl_3$ 4:1	MS, Rose Bengal	395	60	380	87:13	-
1	EtOAc: $CHCl_3$ 4:1	MS, 2 Eq TFA	395	60	380	52:48	5%
1	EtOAc: $CHCl_3$ 4:1	MS, 2 Eq TFA, Chalcone	395	60	380	42:58	5%
1	EtOAc: $CHCl_3$ 4:1	MS, Et_3N	395	90	380	27:73	-
1	EtOAc: $CHCl_3$ 4:1	MS, Chalcone, Malo-	395	90	380	34:66	-

		nitrile					
1	EtOAc: $CHCl_3$ 4:1	MS, Zn^{2+}	395	90	380	84:16	-
**3**	**EtOAc: $CHCl_3$ 4:1**	**MS**	**395**	**90**	**380**	**39:61**	**-**
1	EtOAc: $CHCl_3$ 4:1	MS, β-Nitrostyrene, diethylmalonate	395	90	380	48:52	**Some**
1	EtOAc: MeOH 4:1	MS	395	90	380	Too broad to integrate at 12 ppm	-
1	DCE	MS	395	60	380	50:50	-
1	CH_2Cl_2	MS	395	110	380	50:50	Limited
1	$CHCl_3$	MS	395	70	380	47:53	-
1	EtOAc: $CHCl_3$ 2:3	MS	395	120	380	25:75	-
**1**	**EtOAc: $CHCl_3$ 4:1**	**MS**	**395**	**120**	**380**	**22:78**	**-**
1	EtOAc: CH_2Cl_2 4:1	MS	395	70	380	25:75	-
1	Toluene: CH_2Cl_2 4:1	MS	395	70	380	53:47	-
1	EtOAc: $CHCl_3$ 4:1	MS	395	90	380	30:70	-
1	TCE		395	20	700	47:53	-
1	TCE: CH_2Cl_2:EtOAc 1:1:5		395	20	700	32:68	-
**1**	**CH_2Cl_2:EtOAc 1:4**		**395**	**20**	**700**	**21:79**	**-**

All entries run in 3.5 ml total solvent, with switching ratios obtained after removal of solvent and ^{1}H NMR in d_6-DMSO.

5. Optimisation of thermal switching of catalyst 1

The degree of isomerisation was monitored by following the integral ratio of the *E* and *Z* states of hydrazone proton H(55) (at c. 12 ppm and c. 16 ppm, see characterisation for full numbering).

Initial isomerization of catalyst **1** focused on pure thermal switching. More polar solvents were found to promote isomerization, and pleasing ratios could be obtained in DMF at 90 °C. However, the extended reaction time and high temperature required prompted us to search for effective acid catalysed switching conditions. The addition of TFA to EtOAc allowed virtually quantitative switching after one hour.

Table S2

Entry	*Solvent*	*T/°C*	*Concentration mg/ml*	*Additive*	*Time/ hr*	*Ratio*
**1**	$CHCl_3$	65	0.01	-	3	**25:75**
**2**	EtOAc/DMF 9:1	60	0.01	-	3	**40:60**
**3**	DMF	60	0.01	-	3	**46:54**
**4**	DMSO	60	0.01	-	3	**34:66**
**5**	DMF	90	0.02	-	3	**84:16**
**6**	DMF	90	0.2	-	3	**84:16**
**7**	DMF	90	0.2	-	5	**90:10**
**8**	$CHCl_3$	40	0.02	0.02% TFA	1	**62:38**
**9**	EtOAc	50	0.02	0.04% TFA	1	**83:17**
**10**	EtOAc	50	0.02	0.1% TFA	1	**98:2**

6. Literature Catalysis Examples

Entry	Reagents	Product	Catalyst	Catalyst loading (mol%)	Solvent	Temp.	Time (h)	Conversion (*S*:*R*)
1	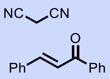	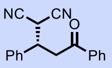	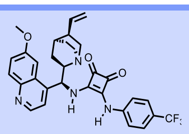	0.5	$CHCl_3$	rt	24	82% (95:5)[S6]
2	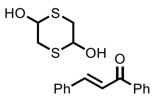	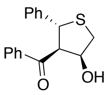	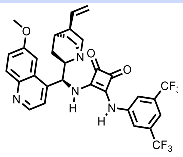	1	Toluene	60•C	6	81% (6:94)[S7]
3	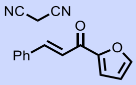	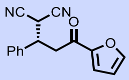	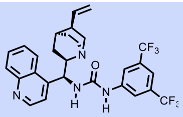	10	*m*-Xylene	rt	18	96%* (93:7)[S8]
4	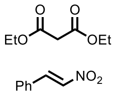	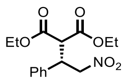	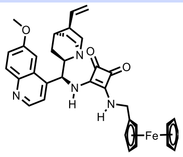	1	CH_2Cl_2	rt	180	83% (95:5)[S9]
5	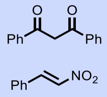	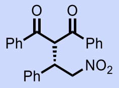	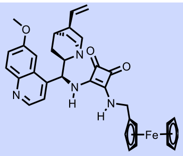	1	CH_2Cl_2	rt	6	89% (95:5)[S9]

7. *in situ* Switching Experiments

Procedure for switching of catalyst state during the course of reaction

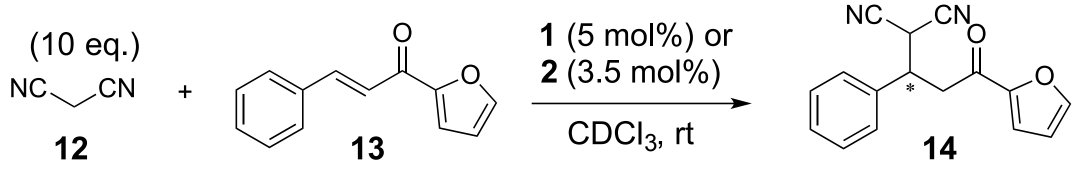

Malonitrile (1.0 mg, 0.015 mmol), (*E*)-1-(furan-2-yl)-3-phenylprop-2-en-1-one (9 mg, 0.045 mmol) and catalyst **2** (0.75 mg, 0.75 µmol) were dissolved in $CDCl_3$ (0.7 ml). Conversion was measured periodically by ^1H NMR spectroscopy and comparison with authentic spectra of starting materials and product. After a given time, the reaction was diluted with 10 ml Et_2O. The precipitate was collected *via* syringe filtration, and dissolved in CH_2Cl_2. The filtrate was stored at − 20 °C for the duration of the switching procedure. The relevant heat or light switch was performed on the catalyst, followed by aqueous extraction with saturated $Na_2CO_{3\ (aq)}$. After drying, the catalyst was recombined with the diluted reaction mixture. Solvent was removed *in vacuo* whilst the flask was cooled to 0 °C. The reaction mixture was immediately dissolved in $CDCl_3$ (0.7 ml) and monitored by NMR. This process was repeated using the alternate switching conditions after a suitable time had passed. No erosion of stereochemistry in the product was observed, as compared to the continuous reaction.

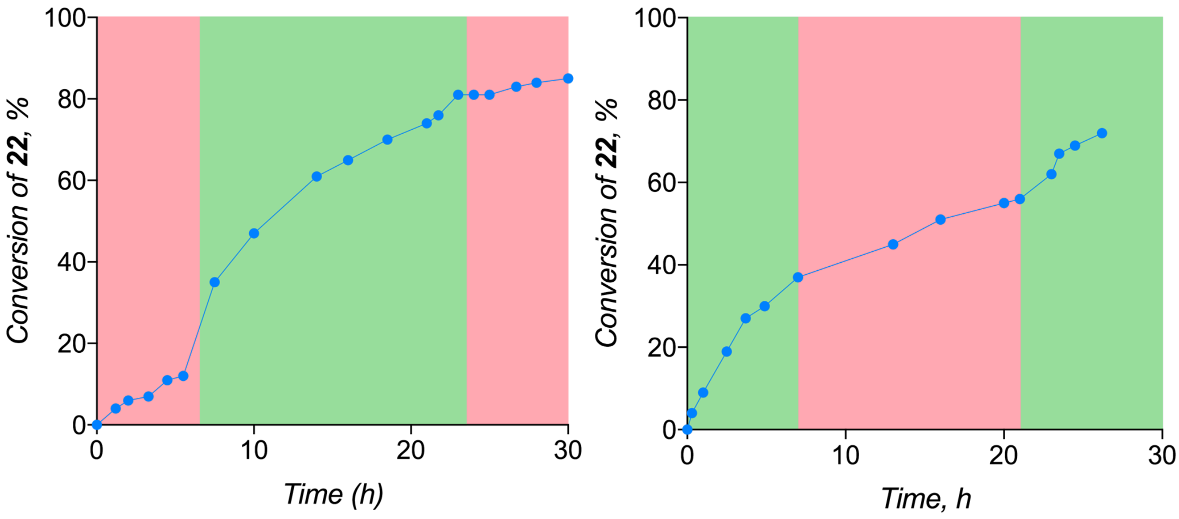

Figure S1. Relative reactivity of ON and OFF states of catalyst **2** towards the addition of malonitrile to chalcone **22** by starting with the OFF state (left) or ON state (right, solid lines are a guide to the eye). A full switching cycle could be carried out during this reaction, starting from either 'ON' or 'OFF' catalyst, using 3.5 mol% of **2** (initial *E*:*Z* ratio 99:1 (ON) or 2:98 (OFF)). After 6 h the *E*-to-Z, or *vice versa*, stimulus was applied (0.1% CF_3CO_2H, 60 min, 50 °C or 395 nm, 700 mW, 20 min) and the reaction continued. After 24 h (for initially OFF **2**) or 21 h (for initially ON **2**) the opposing stimulus was applied reverting catalyst **2** to its initial state.

8. HPLC Traces

Formation of **S6** using catalyst **1** (Chiralpak IC, 90:10 Hexane:*i*PrOH, 1mL/min).

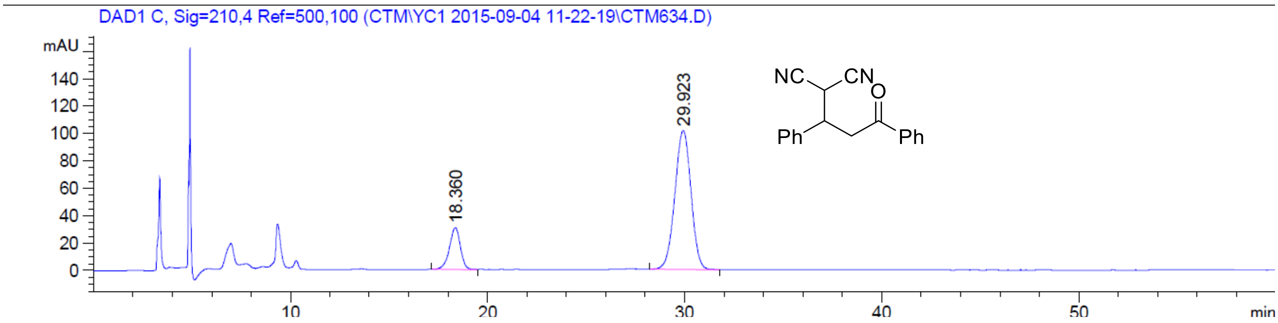

Signal 3: DAD1 C, Sig=210,4 Ref=500,100

Peak #	RetTime [min]	Type	Width [min]	Area [mAU*s]	Height [mAU]	Area %
1	18.360	BB	0.5819	1179.75647	30.59268	16.9746
2	29.923	BB	0.8860	5770.36914	101.39182	83.0254

Formation of **S6** using catalyst **2** (Chiralpak IC, 90:10 Hexane:*i*PrOH, 1mL/min).

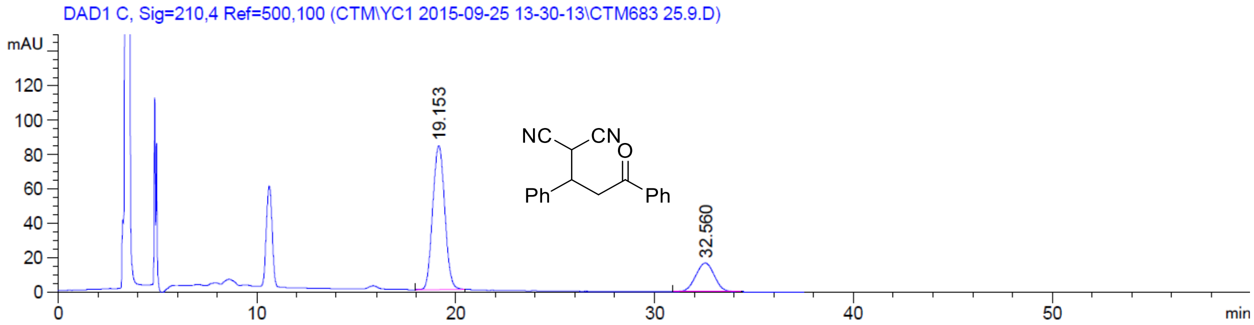

Signal 3: DAD1 C, Sig=210,4 Ref=500,100

Peak #	RetTime [min]	Type	Width [min]	Area [mAU*s]	Height [mAU]	Area %
1	19.155	BB	0.7002	1228.80835	28.09811	77.1590
2	32.989	BB	0.8728	363.75751	5.23420	22.8410

Formation of **S8** using catalyst **1** (Chiralpak IA, 90:10 Hexane:*i*PrOH, 1mL/min).

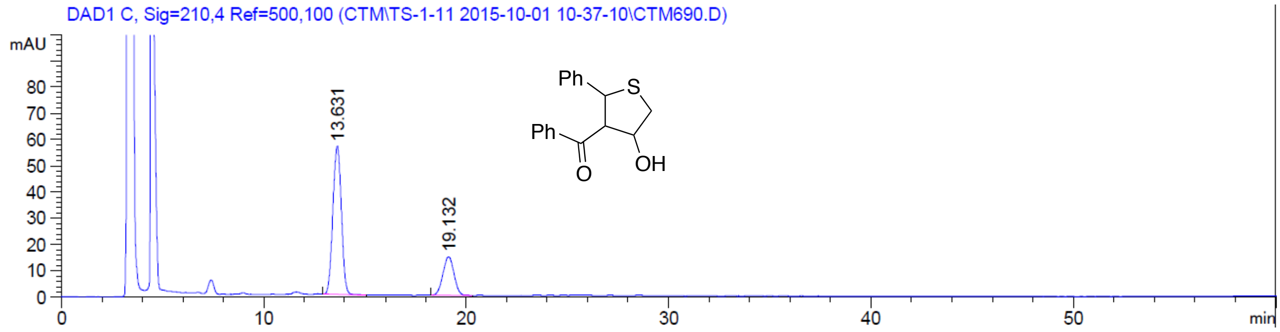

Signal 3: DAD1 C, Sig=210,4 Ref=500,100

Peak #	RetTime [min]	Type	Width [min]	Area [mAU*s]	Height [mAU]	Area %
1	13.631	BB	0.4678	1657.34229	56.57483	74.6692
2	19.132	BB	0.5704	562.23755	14.76148	25.3308

Formation of **S8** using catalyst **2** (Chiralpak IA, 90:10 Hexane:*i*PrOH, 1mL/min).

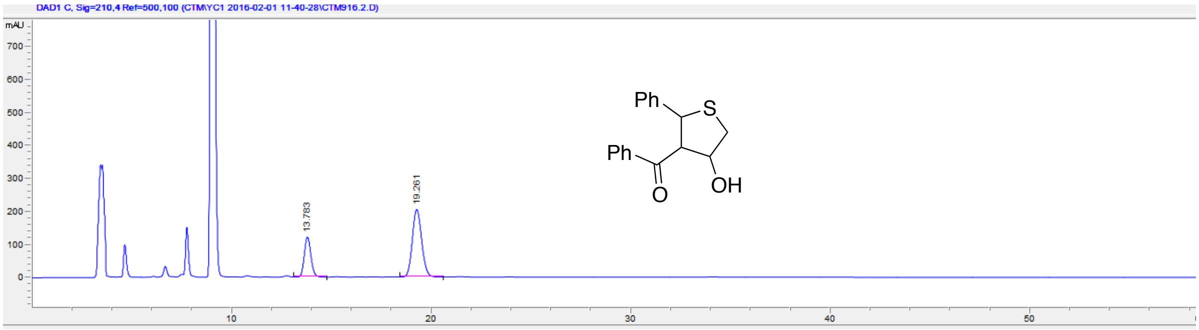

Signal 3: DAD1 C, Sig=210,4 Ref=500,100

Peak #	RetTime [min]	Type	Width [min]	Area [mAU*s]	Height [mAU]	Area %
1	13.783	BB	0.3569	2804.13428	121.64997	29.5406
2	19.261	BB	0.5065	6688.33691	205.05865	70.4594

Formation of **14** using catalyst **1** (Chiralpak IA, 90:10 Hexane:*i*PrOH, 1mL/min).

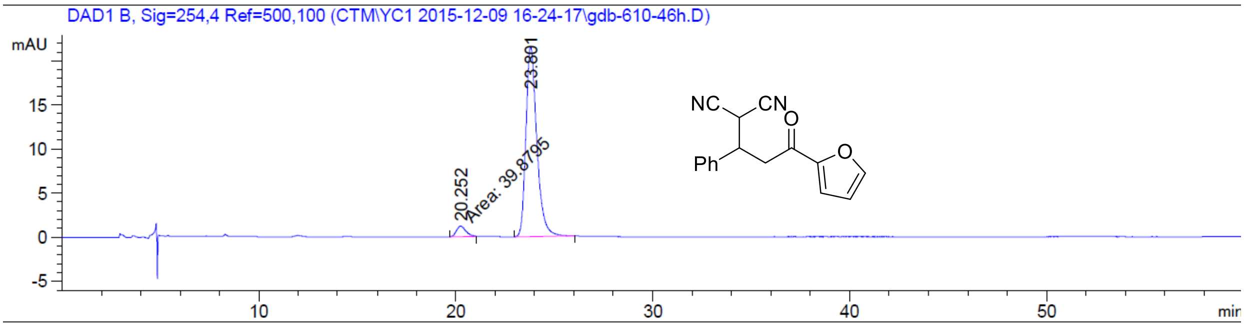

Signal 2: DAD1 B, Sig=254,4 Ref=500,100

Peak #	RetTime [min]	Type	Width [min]	Area [mAU*s]	Height [mAU]	Area %
1	20.252	MM	0.5563	39.87947	1.19485	4.5869
2	23.801	BB	0.5819	829.54236	21.51132	95.4131
Totals :				869.42183	22.70618	

Formation of **14** using catalyst **2** (Chiralpak IA, 90:10 Hexane:*i*PrOH, 1mL/min).

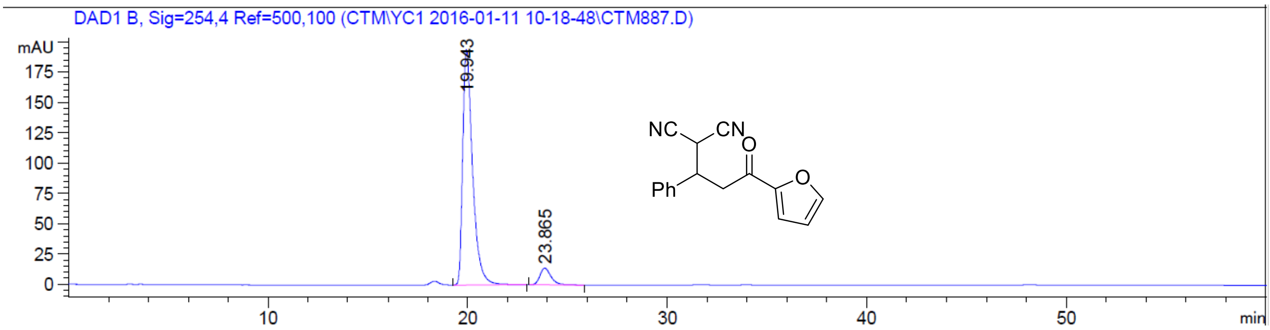

Signal 2: DAD1 B, Sig=254,4 Ref=500,100

Peak #	RetTime [min]	Type	Width [min]	Area [mAU*s]	Height [mAU]	Area %
1	19.943	BB	0.5209	6673.54150	194.21690	92.5924
2	23.865	BB	0.5854	533.89978	13.79792	7.4076
Totals :				7207.44128	208.01483	

Formation of **S10** using catalyst **1** (Chiralpak IC, 90:10 Hexane:*i*PrOH, 1mL/min).

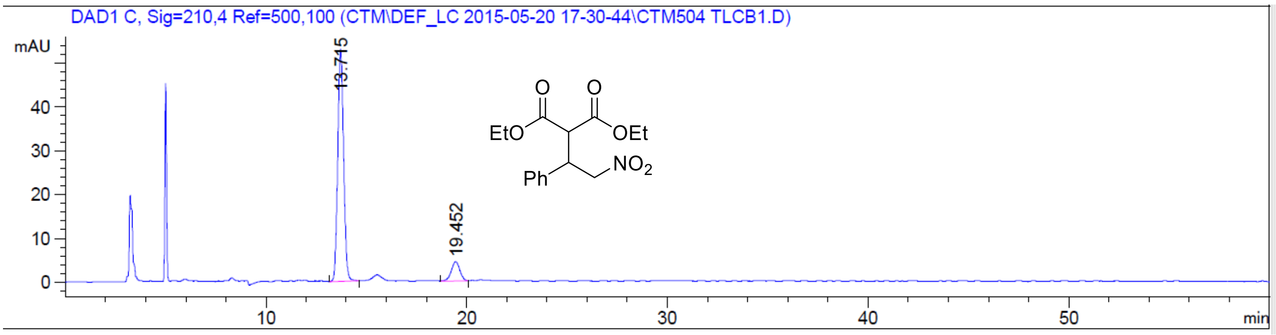

Signal 3: DAD1 C, Sig=210,4 Ref=500,100

Peak #	RetTime [min]	Type	Width [min]	Area [mAU*s]	Height [mAU]	Area %
1	13.715	BB	0.3133	1069.02795	53.00949	89.2687
2	19.452	BB	0.4296	128.51219	4.38877	10.7313

Formation of **S10** using catalyst **2** (Chiralpak IC, 90:10 Hexane:*i*PrOH, 1mL/min).

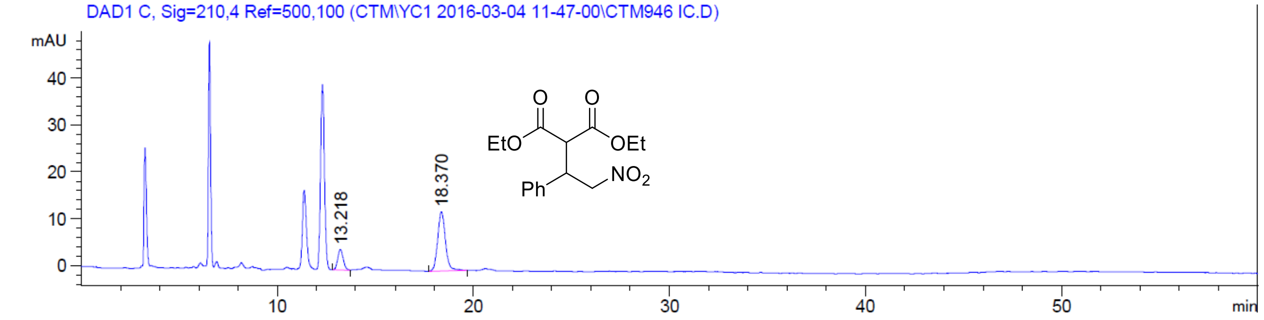

Signal 3: DAD1 C, Sig=210,4 Ref=500,100

Peak #	RetTime [min]	Type	Width [min]	Area [mAU*s]	Height [mAU]	Area %
1	13.218	BB	0.3000	85.15978	4.39684	19.6072
2	18.370	BB	0.4224	349.16968	12.63498	80.3928

Formation of **S13** using catalyst **1** (Chiralpak IA, 90:10 Hexane:*i*PrOH, 1mL/min).

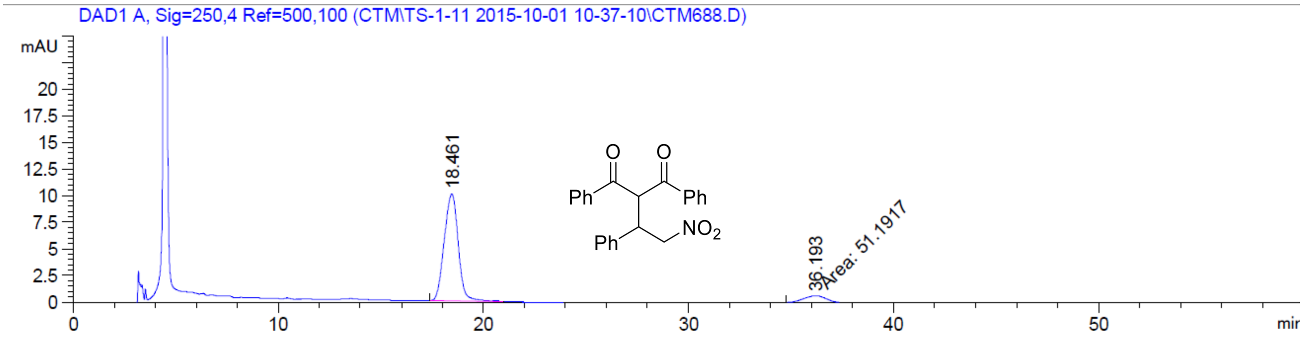

Signal 1: DAD1 A, Sig=250,4 Ref=500,100

Peak #	RetTime [min]	Type	Width [min]	Area [mAU*s]	Height [mAU]	Area %
1	18.461	BB	0.7091	478.63751	10.05877	90.5574
2	36.193	MM	1.2775	49.90842	6.51126e-1	9.4426

Formation of **S13** using catalyst **2** (Chiralpak IA, 90:10 Hexane:*i*PrOH, 1mL/min).

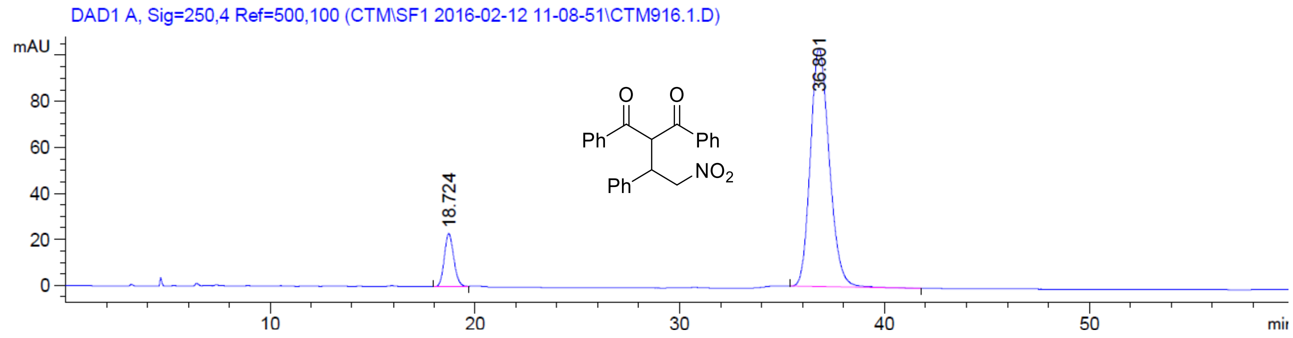

Signal 1: DAD1 A, Sig=250,4 Ref=500,100

Peak #	RetTime [min]	Type	Width [min]	Area [mAU*s]	Height [mAU]	Area %
1	18.724	BB	0.4944	738.75970	23.02057	9.9869
2	36.801	BB	0.9923	6658.49951	103.54144	90.0131

Formation of **S15** using catalyst **1** (Chiralpak IA, 90:10 Hexane:*i*PrOH, 1mL/min).

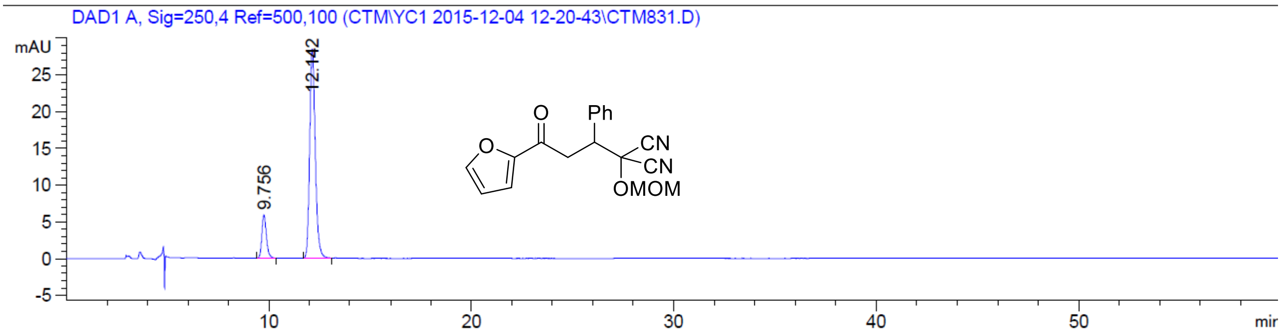

Signal 1: DAD1 A, Sig=250,4 Ref=500,100

Peak #	RetTime [min]	Type	Width [min]	Area [mAU*s]	Height [mAU]	Area %
1	9.756	BB	0.2392	91.24355	5.87517	14.3406
2	12.142	BB	0.2937	545.01532	28.41956	85.6594

Formation of **S15** using catalyst **2** (Chiralpak IA, 90:10 Hexane:*i*PrOH, 1mL/min).

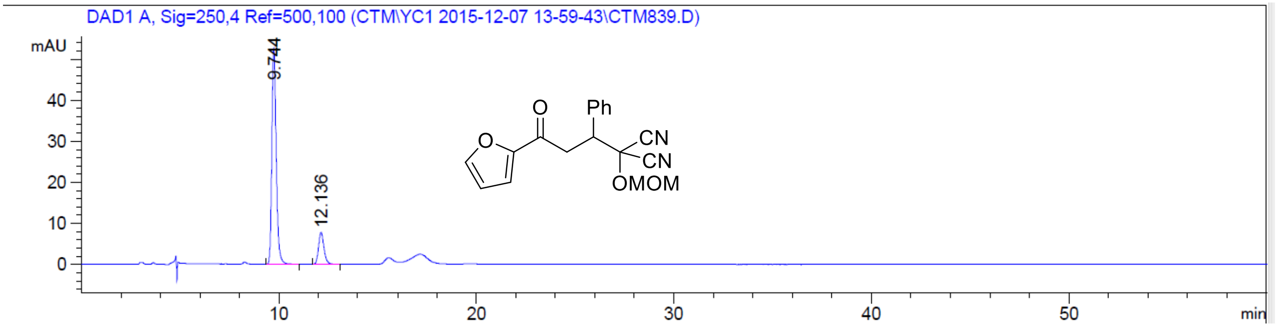

Signal 1: DAD1 A, Sig=250,4 Ref=500,100

Peak #	RetTime [min]	Type	Width [min]	Area [mAU*s]	Height [mAU]	Area %
1	9.744	BB	0.2353	809.28400	52.65082	84.5287
2	12.136	BB	0.2904	148.12373	7.77182	15.4713

9. NMR Spectra

Spectra of intermediate **S1**

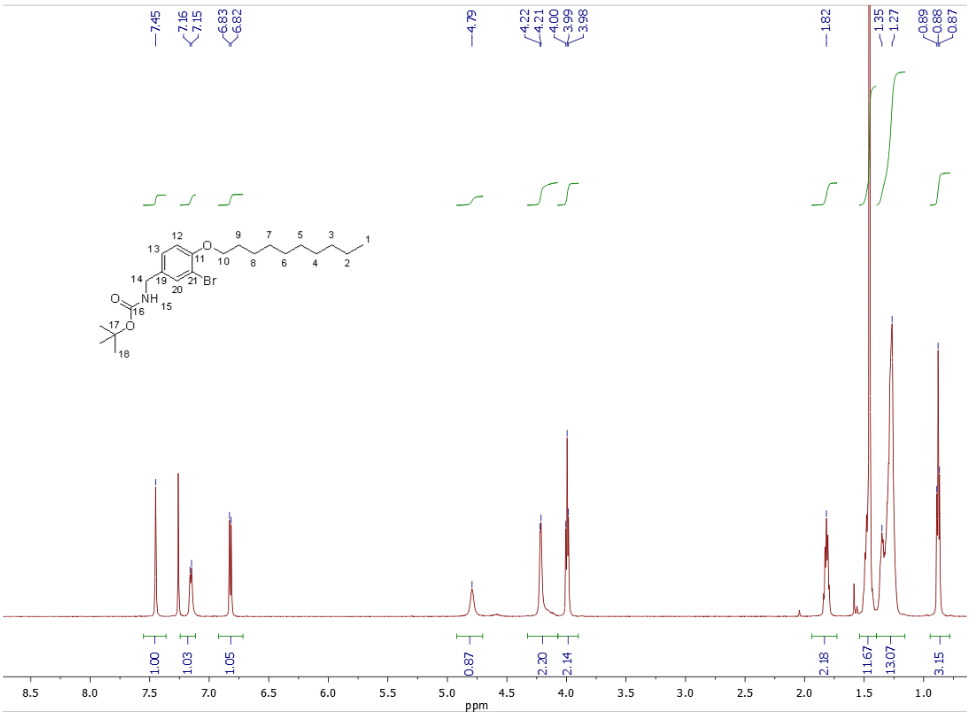

^{1}H NMR (600 MHz, $CDCl_3$) of intermediate **S1**

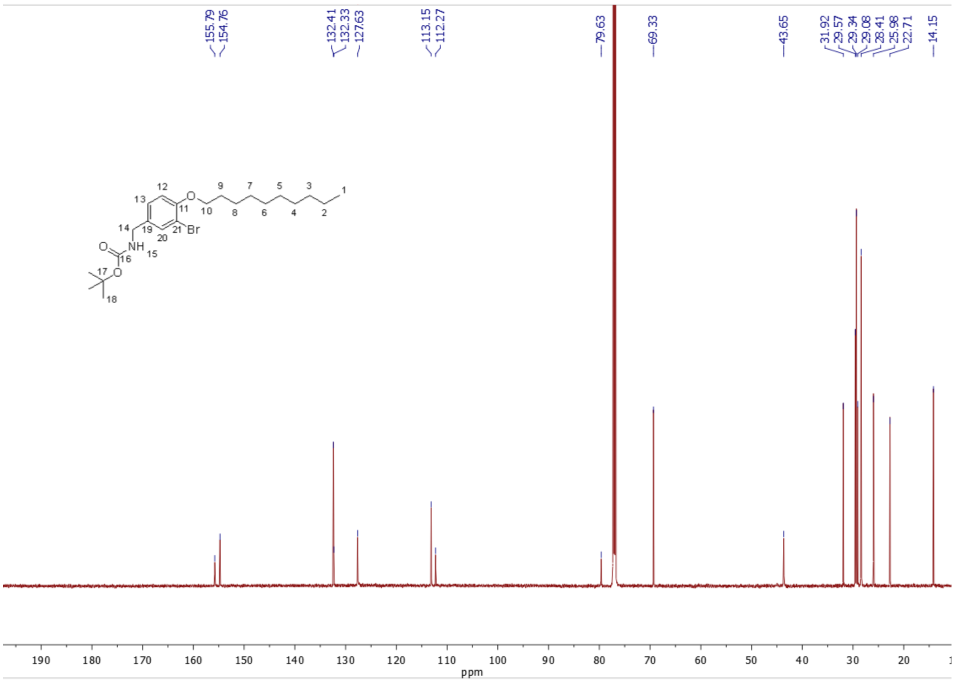

^{13}C NMR (150 MHz, $CDCl_3$) of intermediate **S1**

NMR spectra of intermediate **S2**

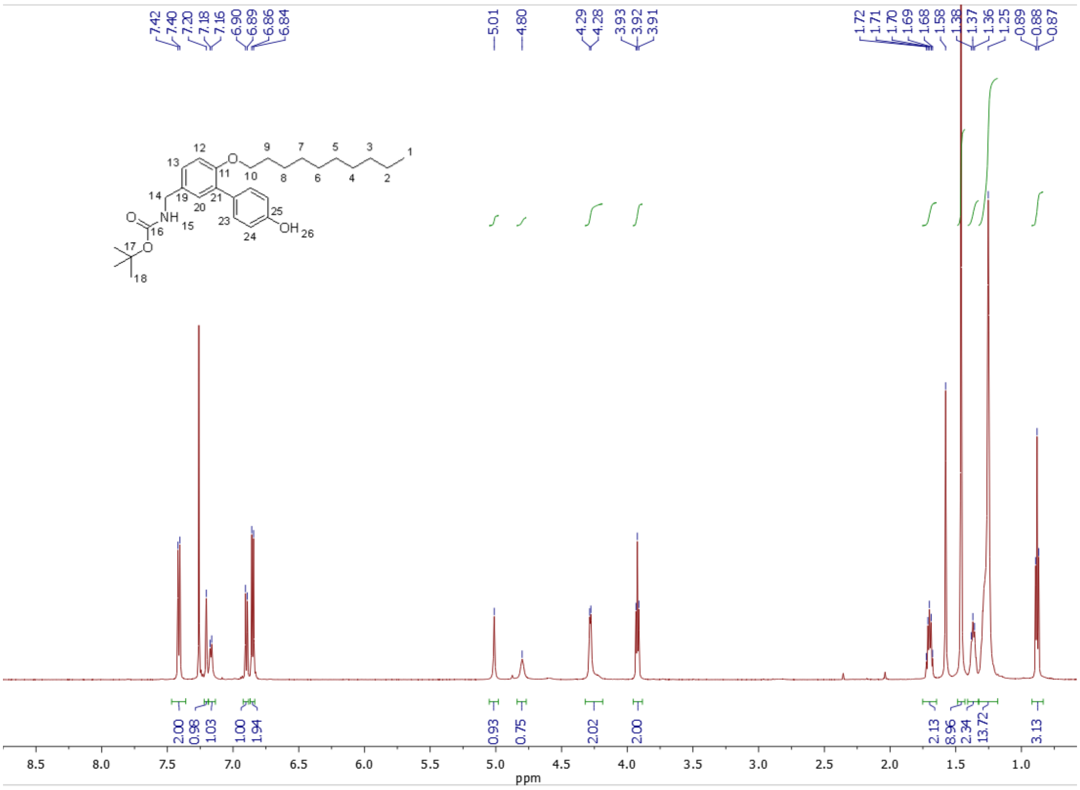

^{1}H NMR (600 MHz, $CDCl_3$) of intermediate **S2**

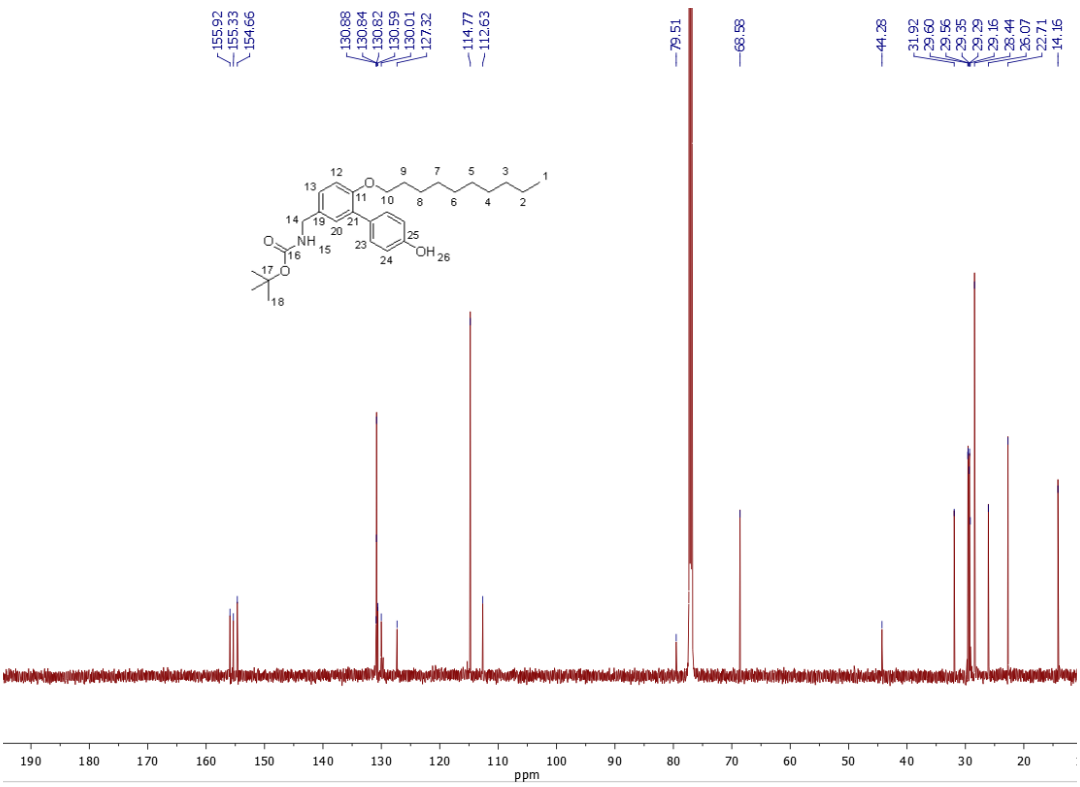

^{13}C NMR (150 MHz, $CDCl_3$) of intermediate **S2**

NMR spectra of intermediate **S3**

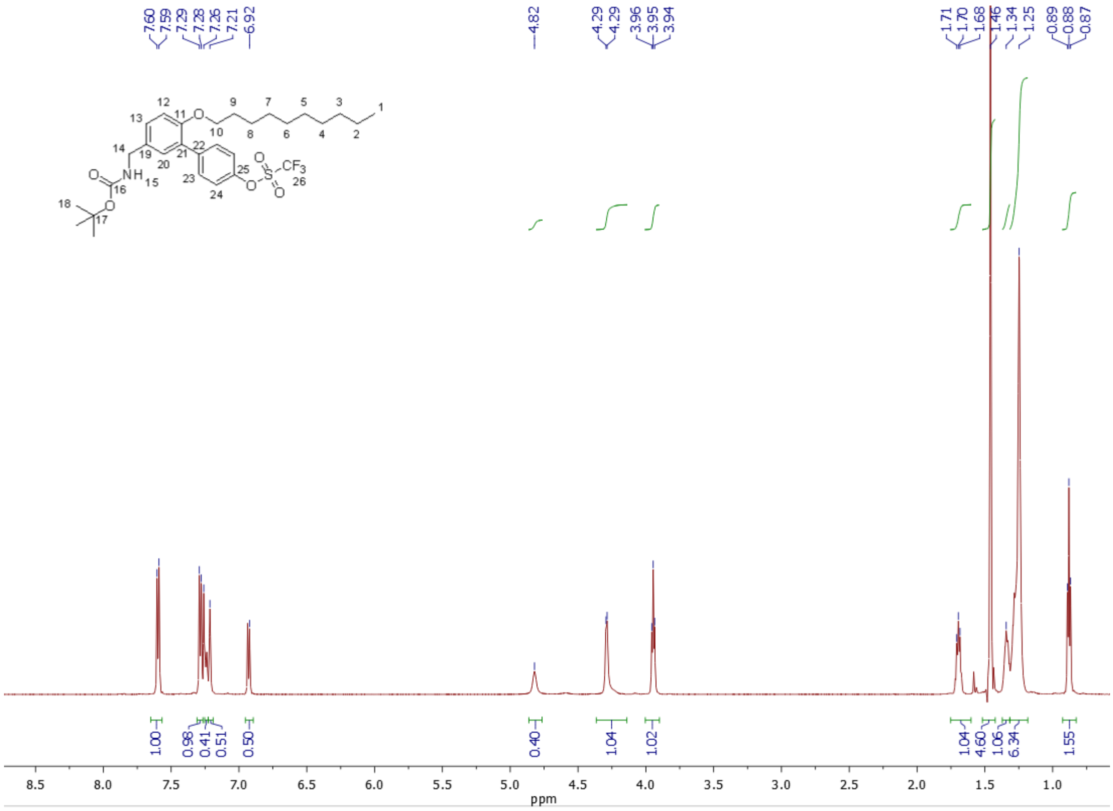

^{1}H NMR (600 MHz, CD_2Cl_2) of intermediate **S3**

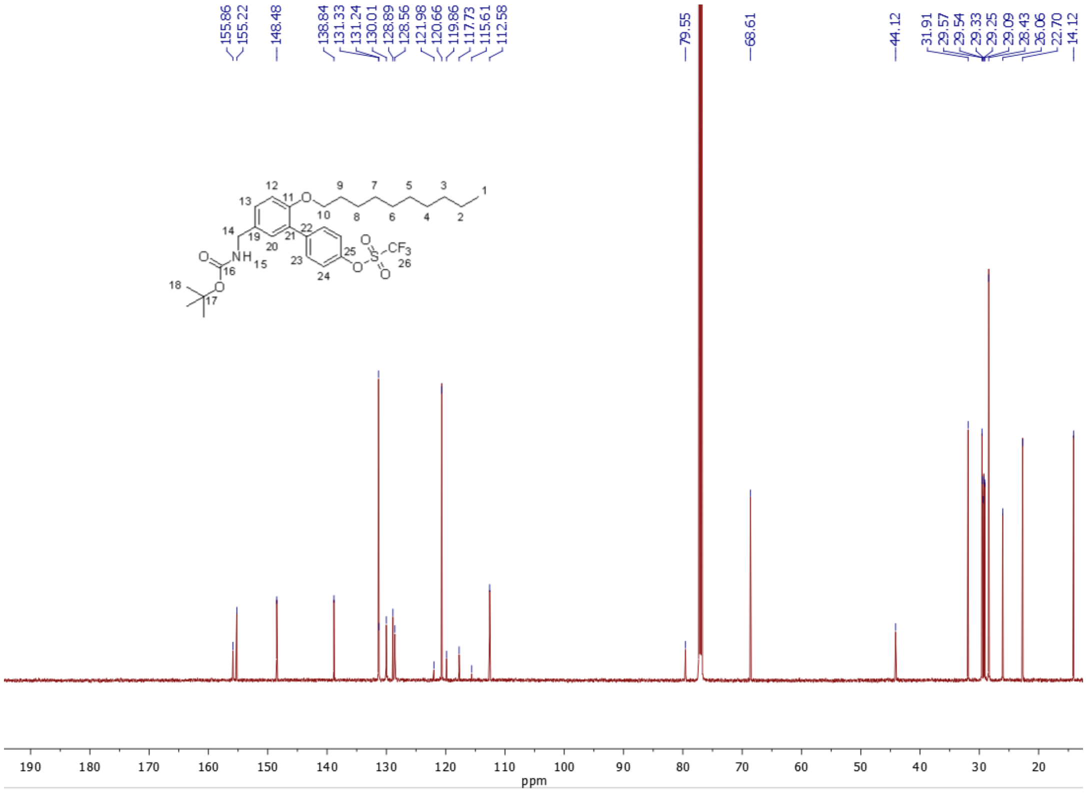

^{13}C NMR (150 MHz, CD_2Cl_2) of intermediate **S3**

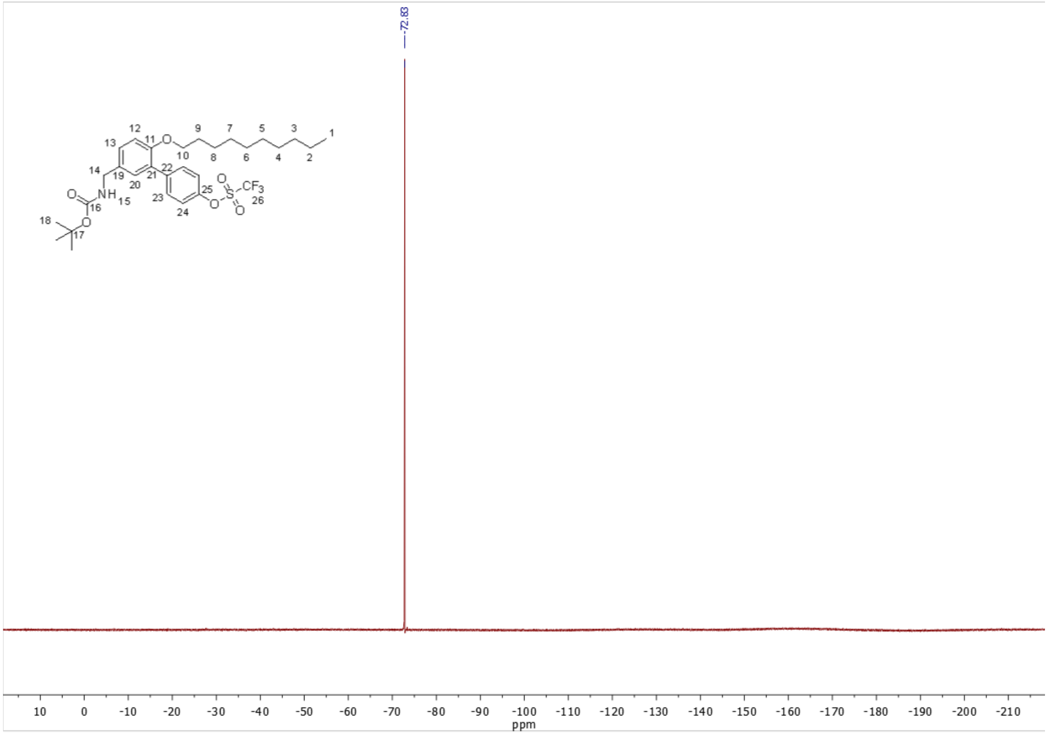

^{19}F NMR (376 MHz, $CDCl_3$) of intermediate **S3**

NMR spectra of intermediate **3**

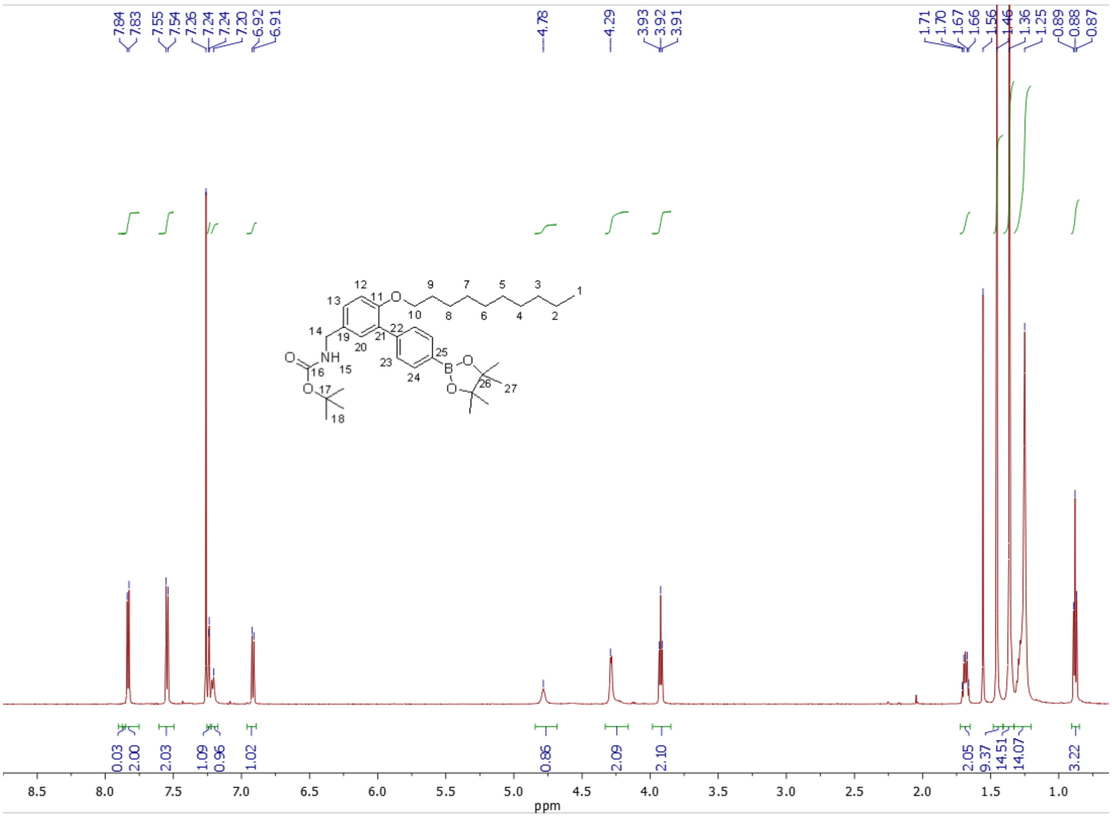

^{1}H NMR (600 MHz, $CDCl_3$) of intermediate **3**

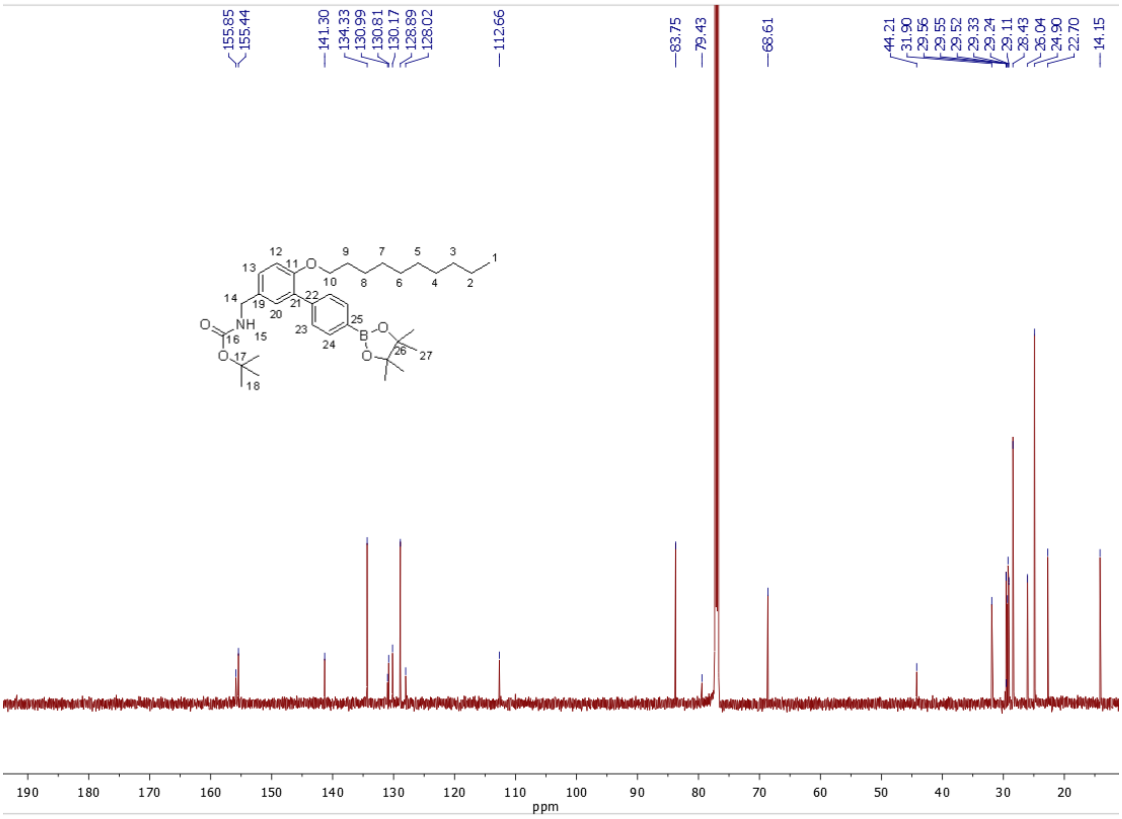

^{13}C NMR (150 MHz, $CDCl_3$) of intermediate **3**

NMR spectra of intermediate **S4**

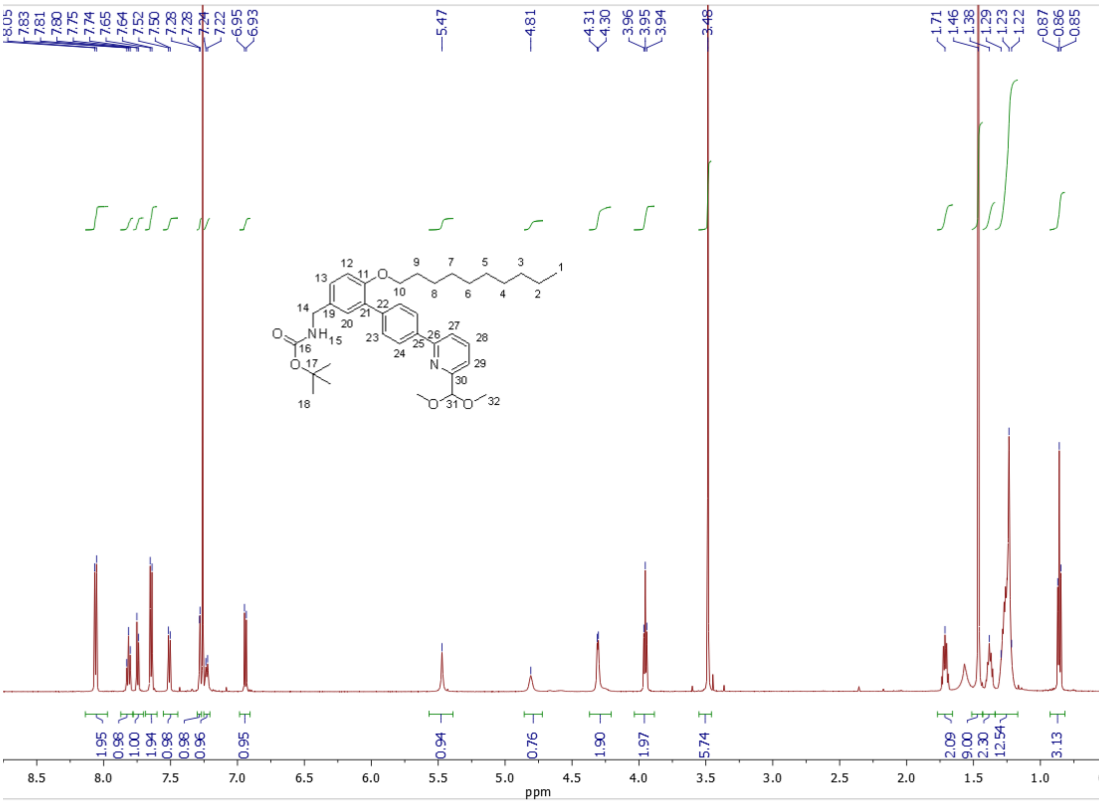

^{1}H NMR (600 MHz, $CDCl_3$) of intermediate **S4**

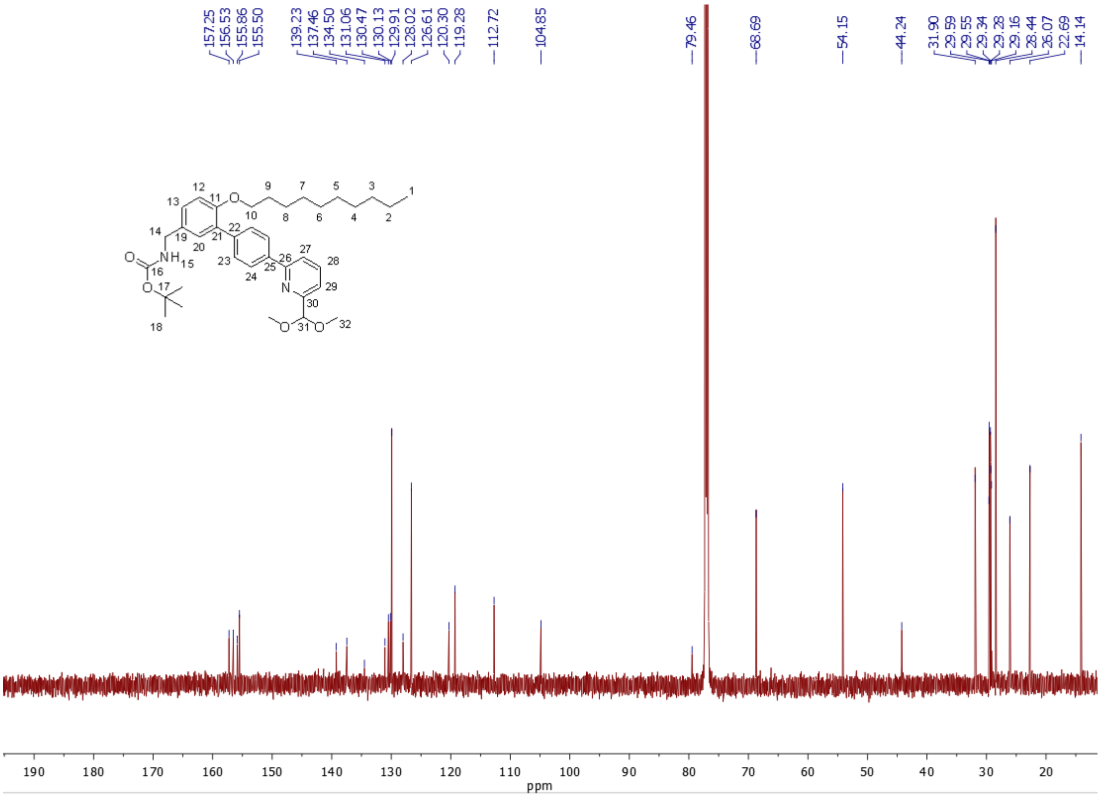

^{13}C NMR (150 MHz, $CDCl_3$) of intermediate S**4**

NMR spectra of intermediate **9**

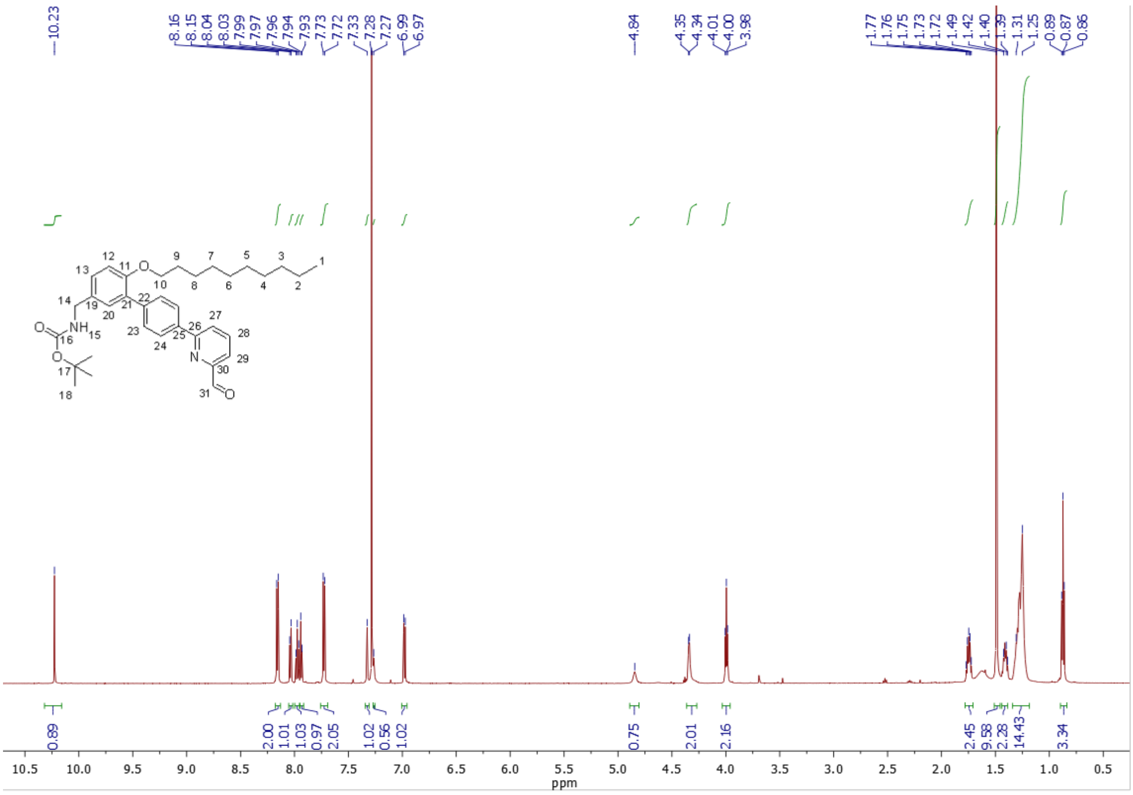

^{1}H NMR (600 MHz, $CDCl_3$) of intermediate **9**

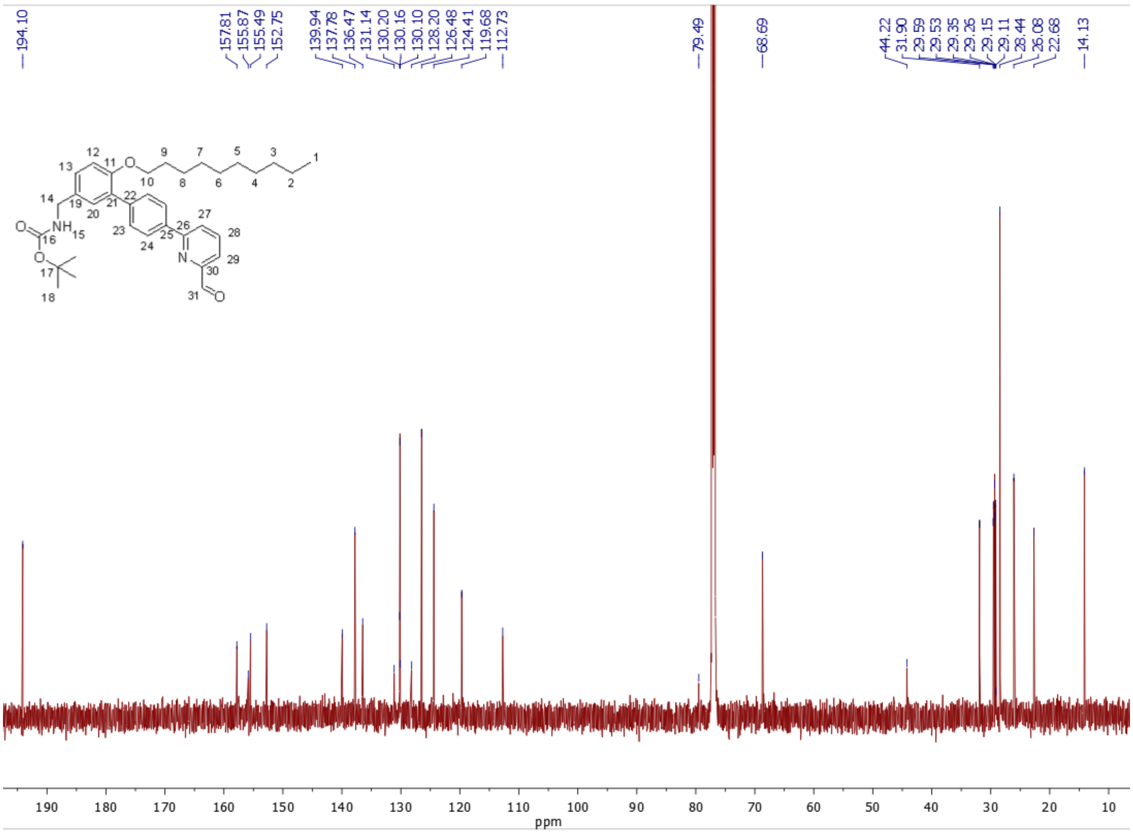

^{13}C NMR (150 MHz, $CDCl_3$) of intermediate **9**

NMR spectra of intermediate **11**

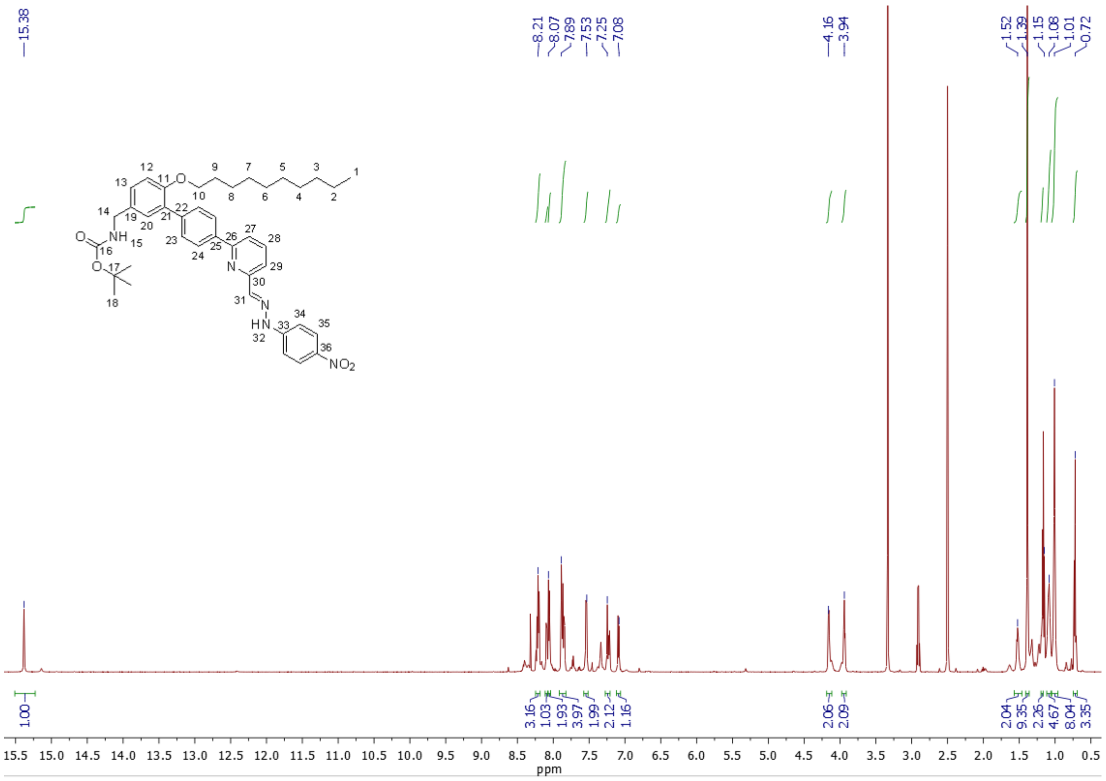

^{1}H NMR (600 MHz, d_6–DMSO) of intermediate **11**

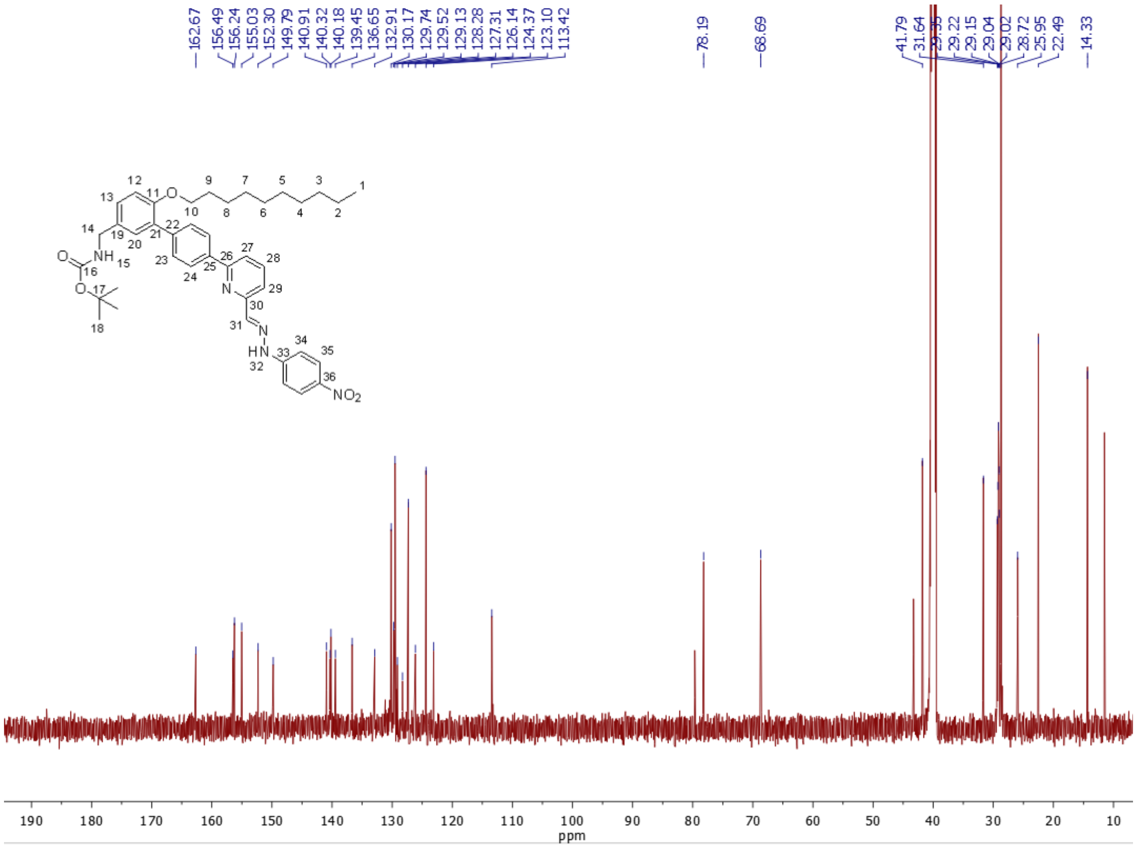

^{13}C NMR (150 MHz, d_6–DMSO) of intermediate **11**

NMR spectra of bifunctional catalyst **1**

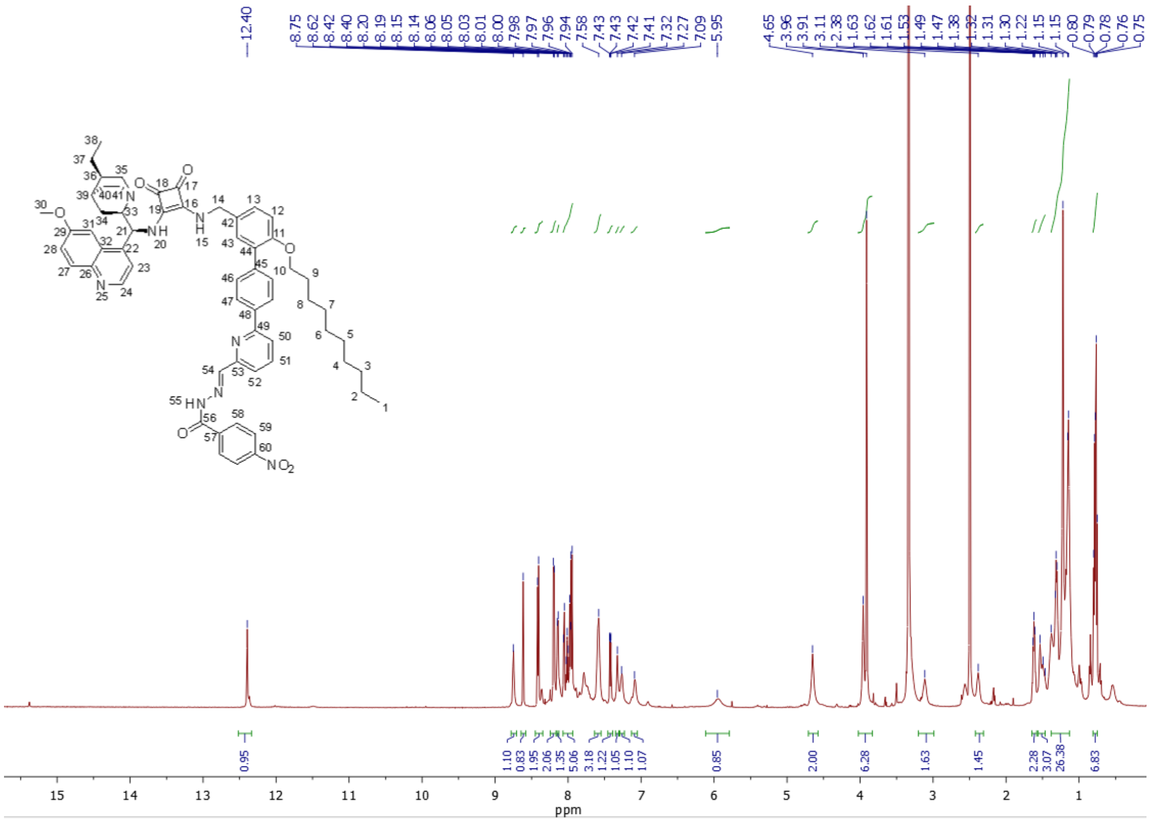

^{1}H NMR (600 MHz, d_6–DMSO) of bifunctional catalyst **1**

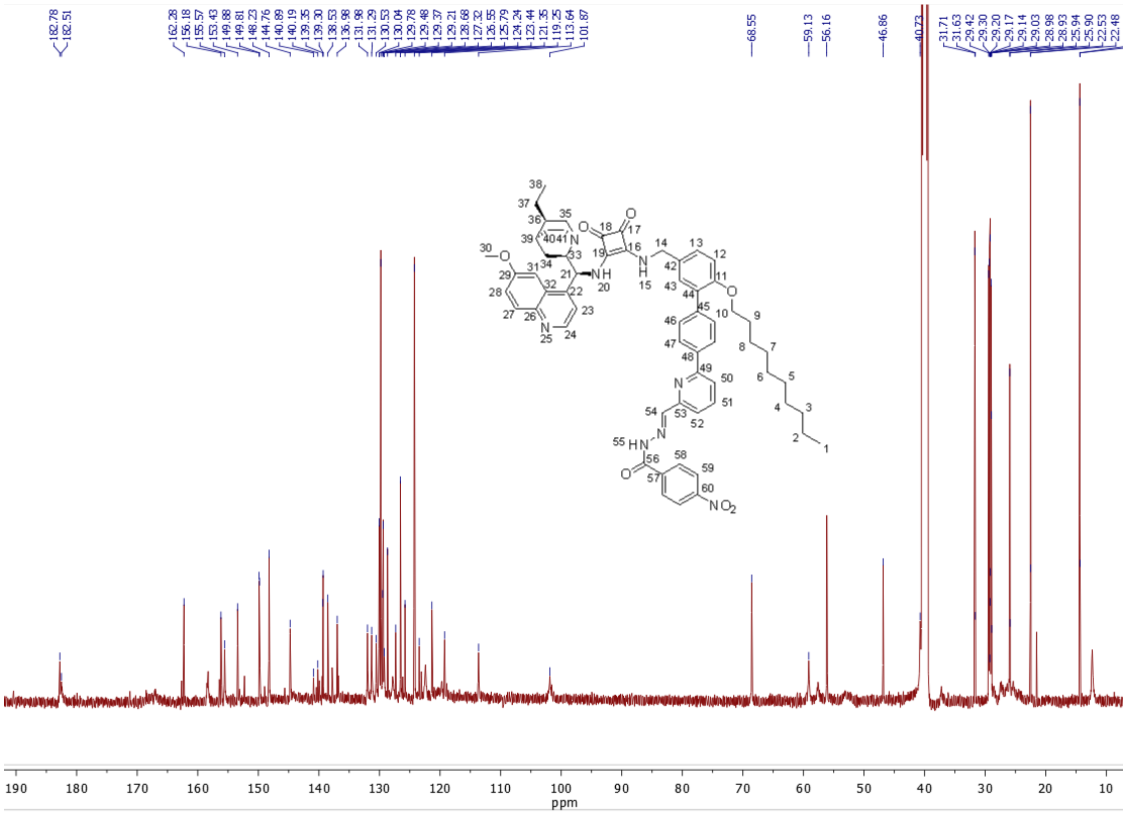

^{13}C NMR (150 MHz, d_6–DMSO) of bifunctional catalyst **1**

NMR spectra of intermediate **S5**

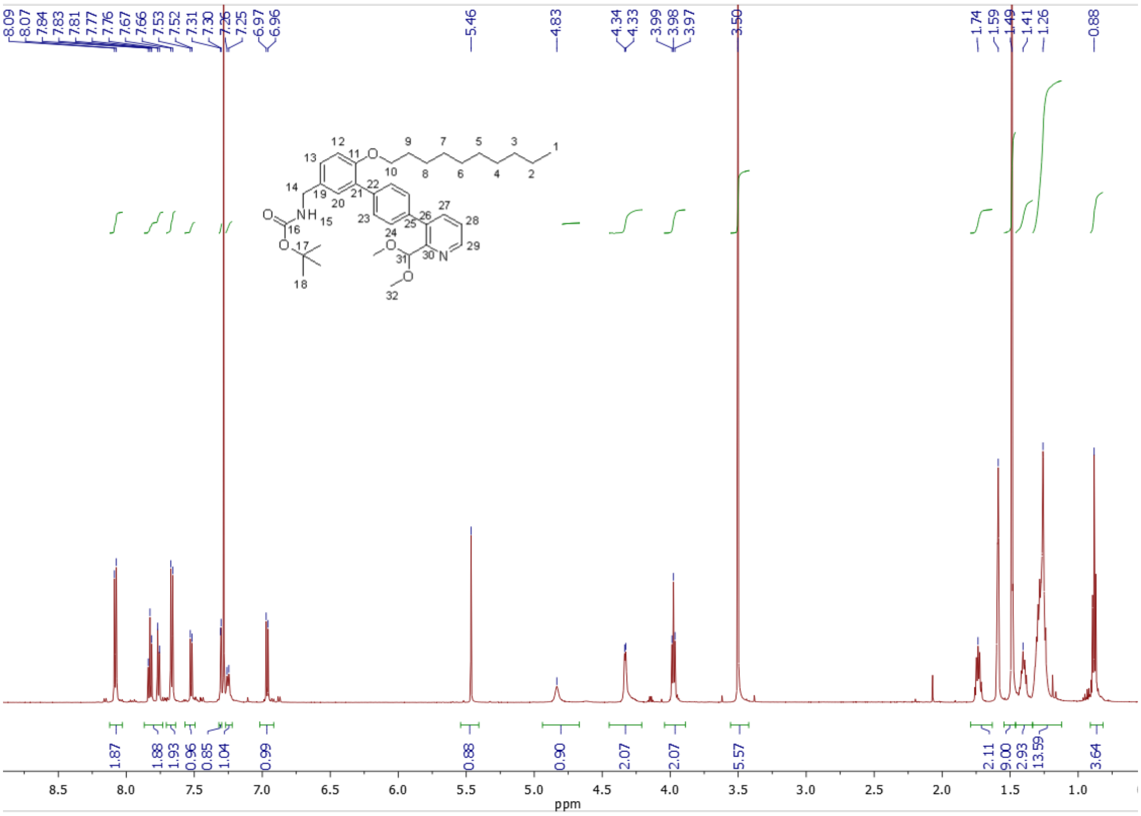

^{1}H NMR (600 MHz, $CDCl_3$) of intermediate **S5**

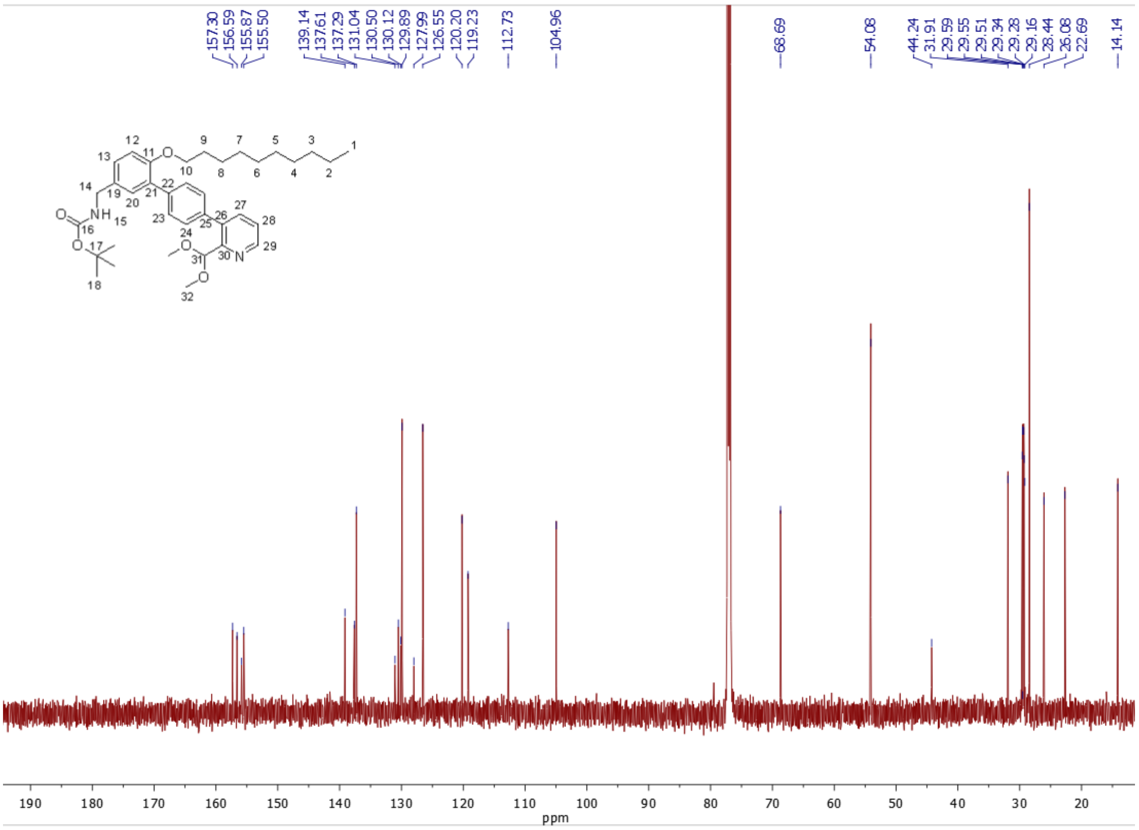

^{13}C NMR (150 MHz, $CDCl_3$) of intermediate **S5**

NMR spectra of intermediate **8**

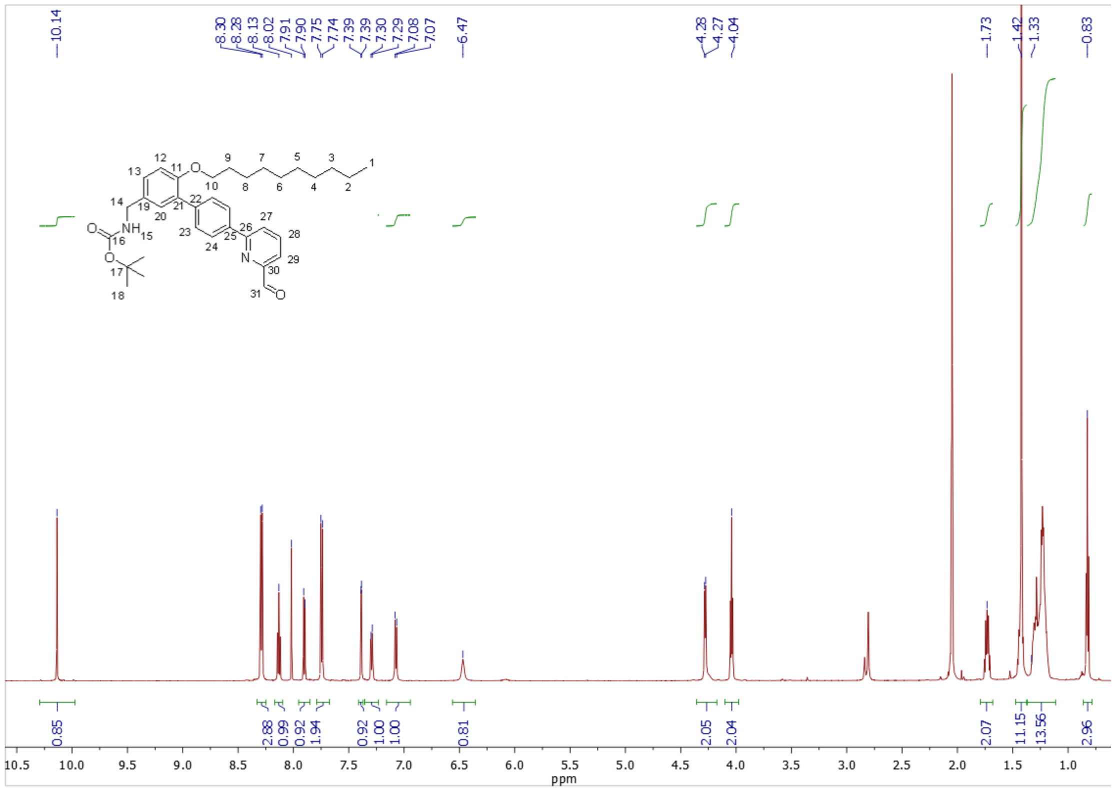

^{1}H NMR (600 MHz, d_6–Acetone) of intermediate **8**

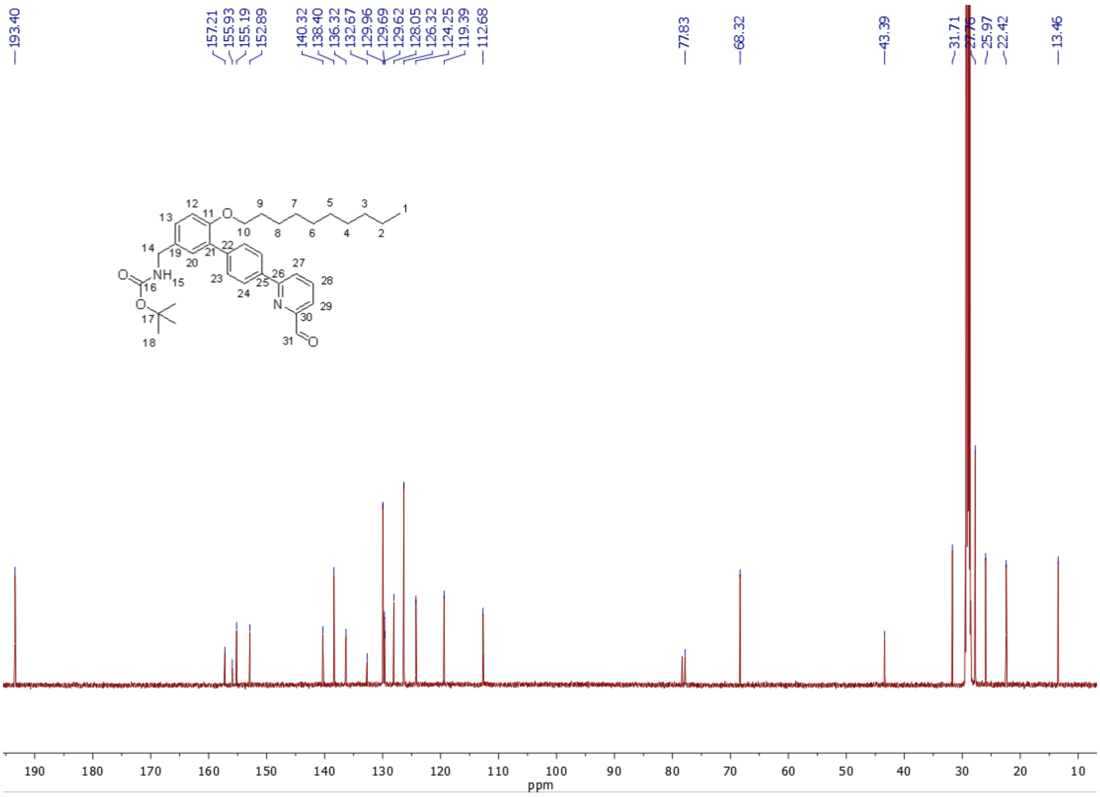

^{13}C NMR (150 MHz, d_6–Acetone) of intermediate **8**

NMR spectra of intermediate **10**

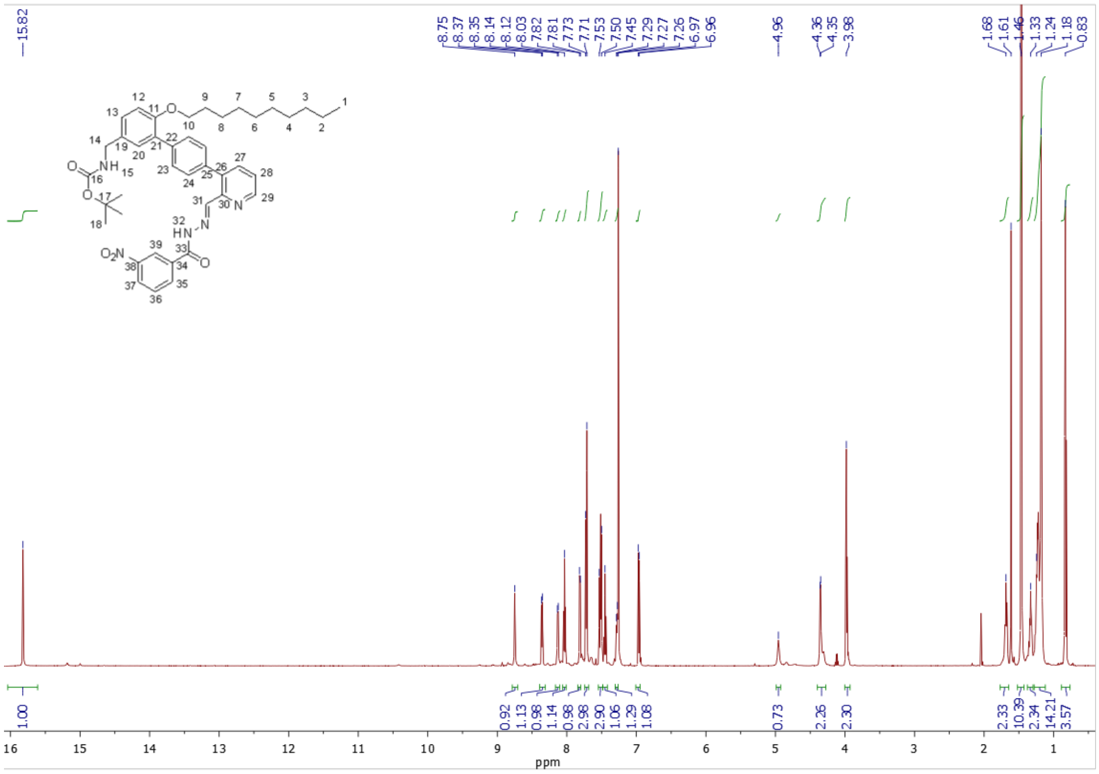

^{1}H NMR (600 MHz, CDCl$_3$) of intermediate **10**

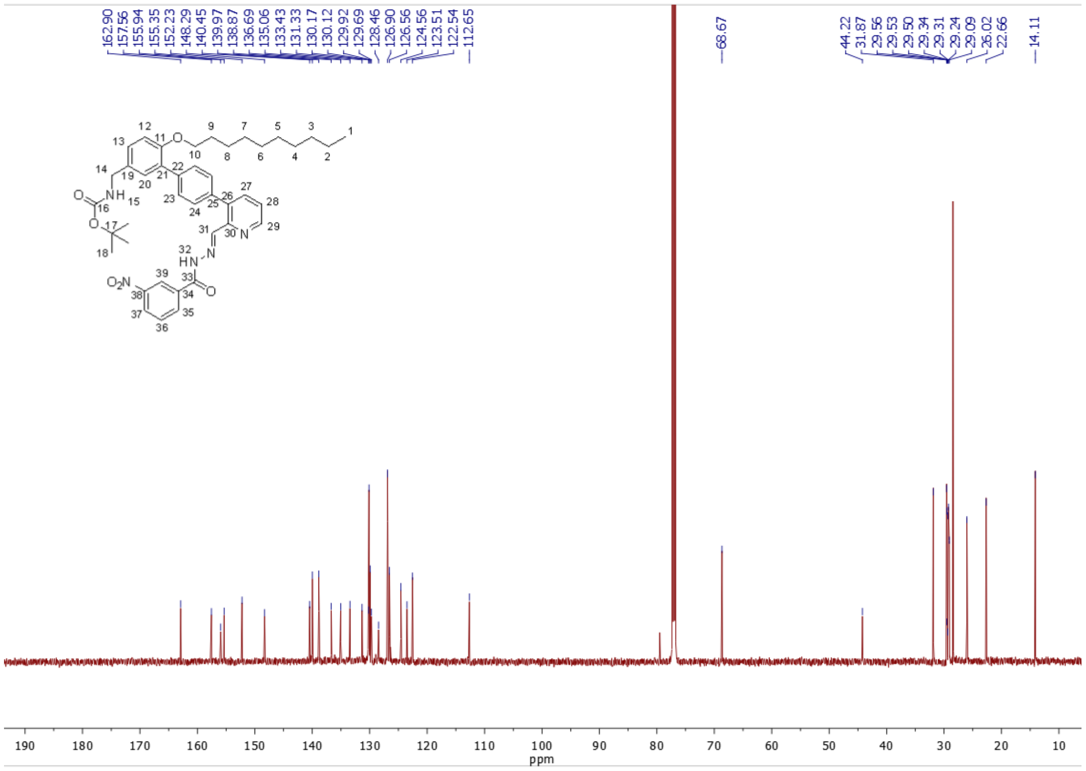

^{13}C NMR (150 MHz, CDCl$_3$) of intermediate **10**

NMR spectra of bifunctional catalyst **2**

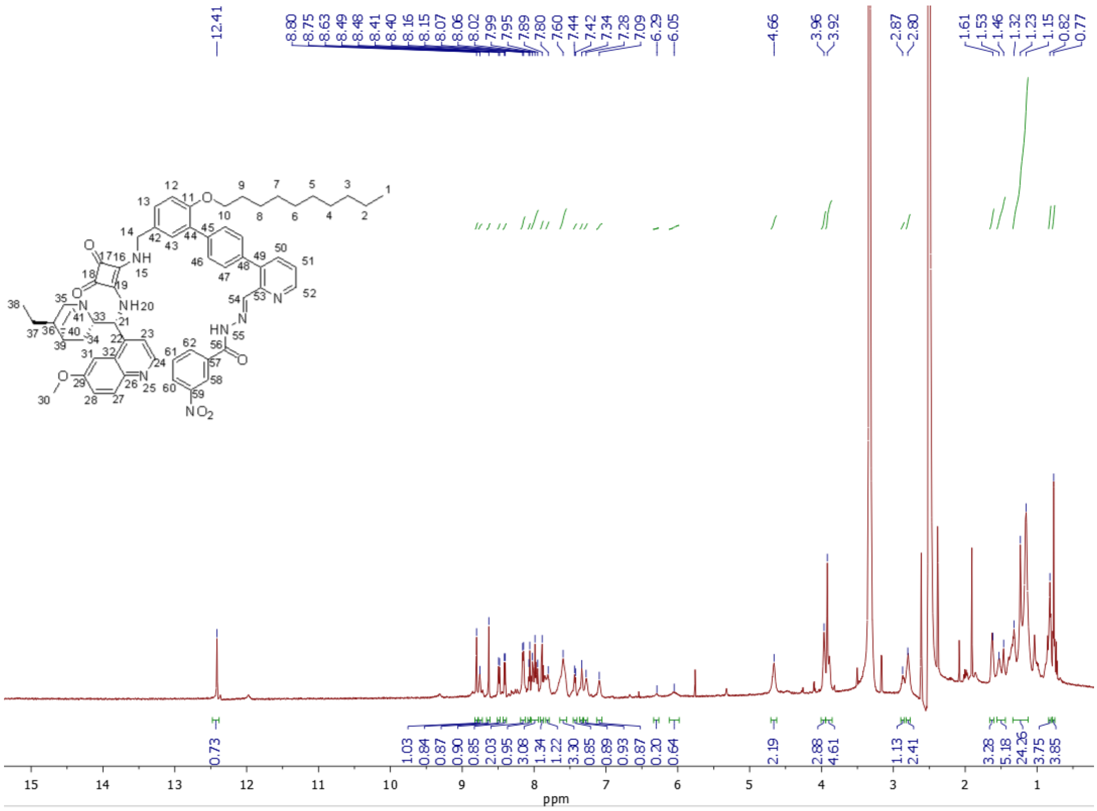

^{1}H NMR (600 MHz, d_6–DMSO) of bifunctional catalyst **2**

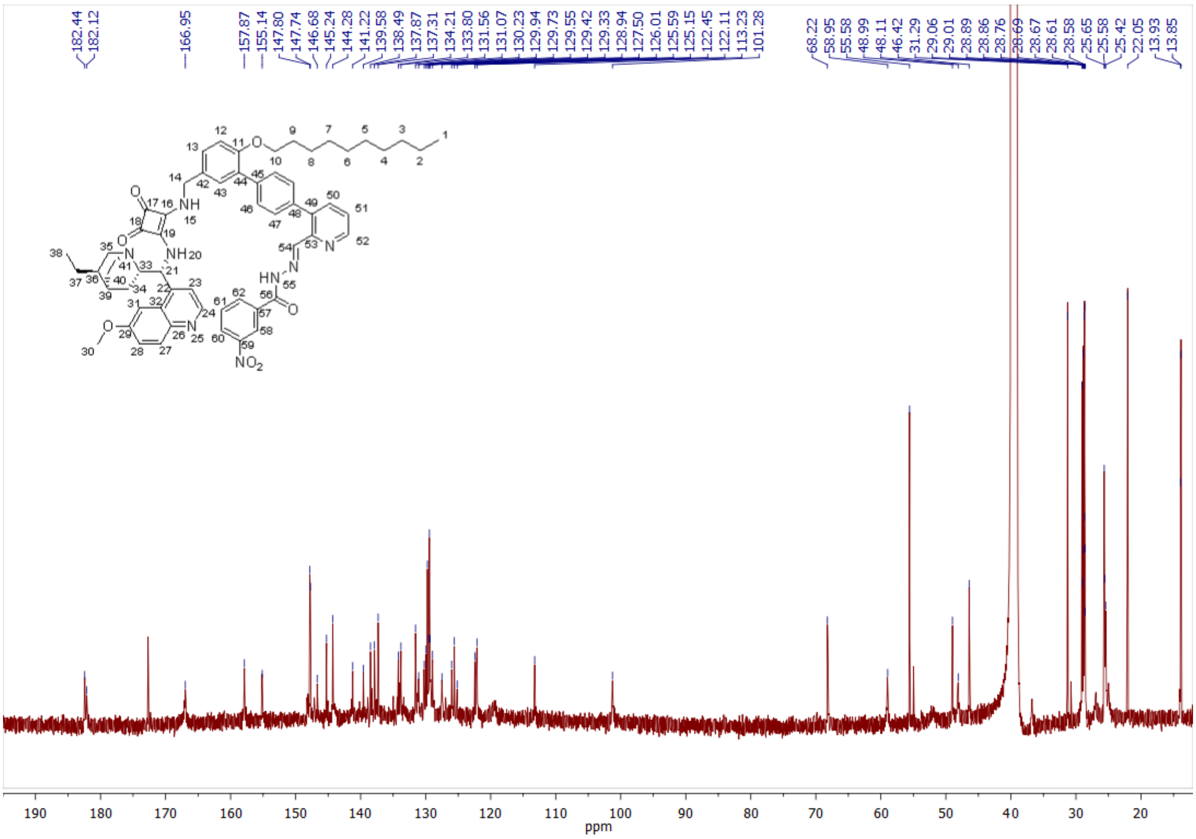

^{13}C NMR (150 MHz, d_6–DMSO) of bifunctional catalyst **2**

NMR spectra of addition product **S15**

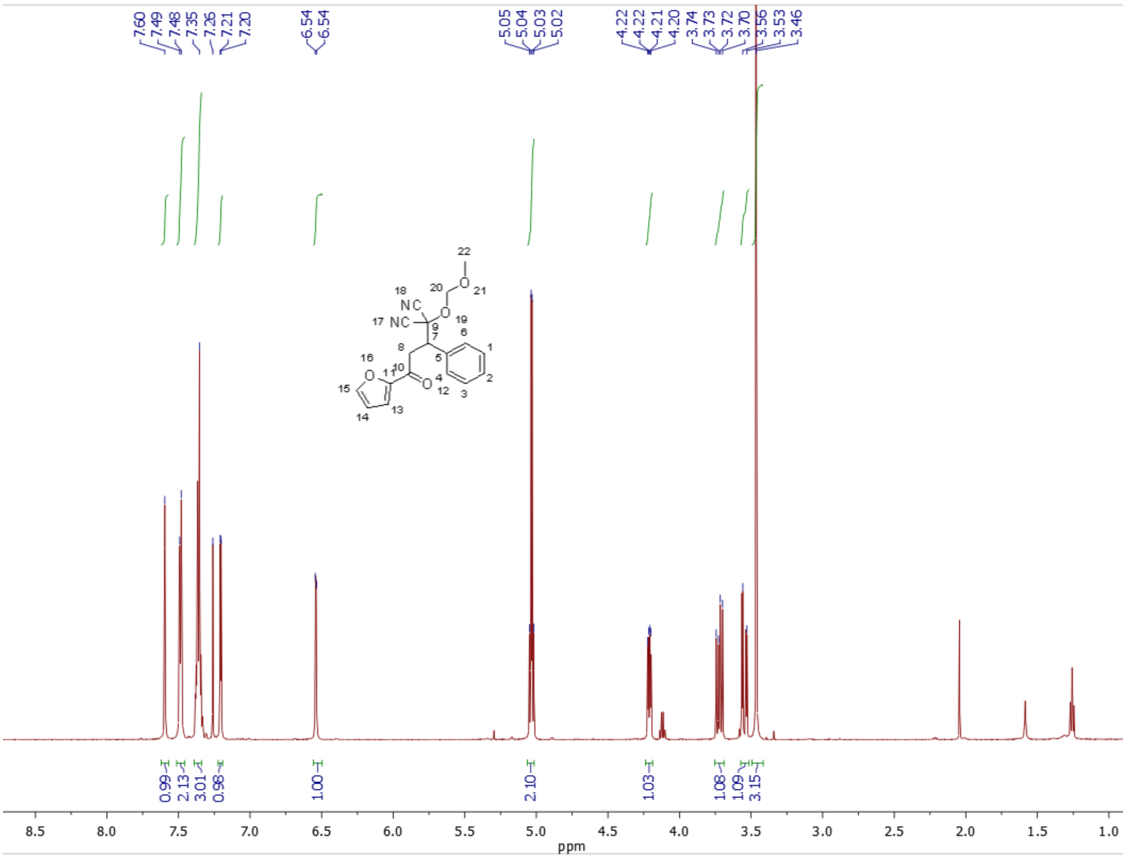

^{1}H NMR (600 MHz, CDCl$_3$) of addition product **S15**

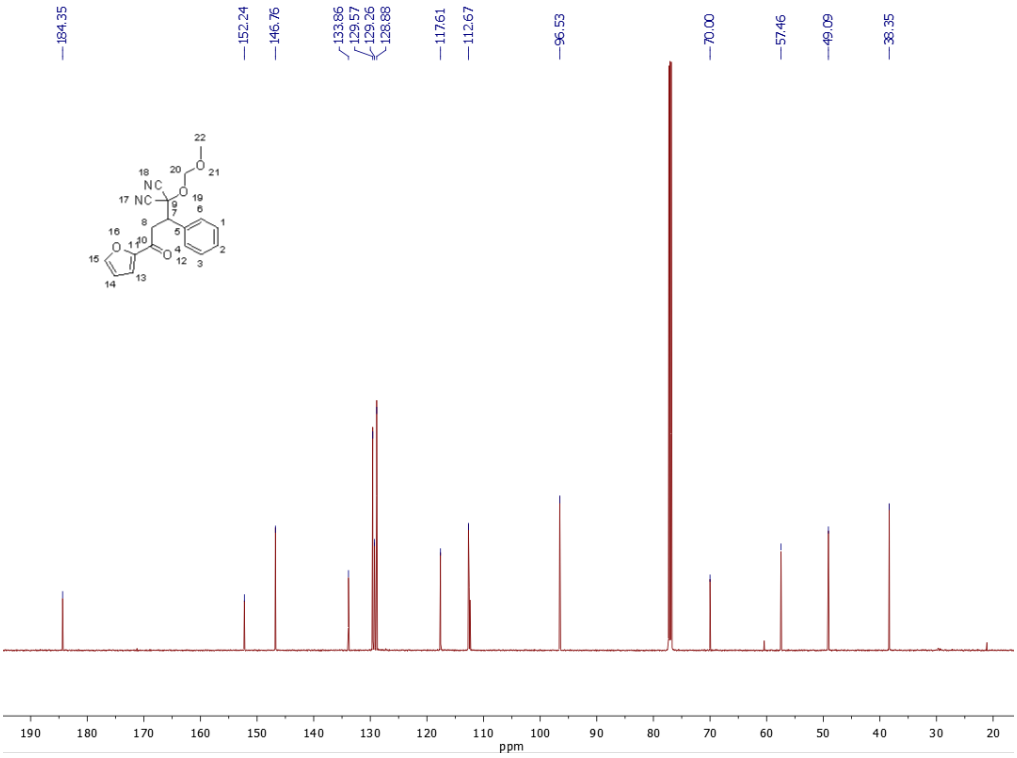

^{13}C NMR (150 MHz, CDCl$_3$) of addition product **S15**

10. References

(S1) R. G. Hicks, B. D. Koivisto and M. T. Lemaire, *Org. Lett.* 2004, **6**, 1887.

(S2) T. Kuroda, M. Takahashi, T. Ogiku, H. Ohmizu, T. Nishitani, K. Kondo and T. Iwasaki, *J. Org. Chem.* 1994, **59**, 7353.

(S3) C. Cassani, R. Martín-Rapun, E. Arceo, F. Bravo and P. Melchiorre, *Nat. Protoc.* 2013, **8**, 325.

(S4) K. Gobis, H. Foks, E. Augustynewicz-Kopec, M. Szczesio, A. Olczak and M. L. Glouka, *Monatsh. Chem.* 2012, **143**, 607.

(S5) T. Curtis and G. Struve, *J. Prakt. Chem.* 1894, **50**, 295.

(S6) W. Yang, Y. Jiu and D.-M. Du, *Org. Biomol. Chem.* 2012, **10**, 332.

(S7) J.-B. Ling, Y. Su, H.-L. Zhu, G.-Y. Wang and P.-F. Xu, *Org. Lett.* 2012, **14**, 1090.

(S8) N. Molleti, K. N. Rana and V. K. Singh, *Org. Lett.* 2012, **14**, 4322.

(S9) K. S. Rao, R. Trivedi and M. L. Kantam, *Synlett* 2015, **26**, 221.

(S10) K. S. Yang, A. E. Nibbs, Y. E. Türkmen and V. H. Rawal, *J. Am. Chem. Soc.* 2013, **135**, 16050.
